# Bottleneck size and selection level reproducibly impact evolution of antibiotic resistance

**DOI:** 10.1038/s41559-021-01511-2

**Published:** 2021-07-26

**Authors:** Niels Mahrt, Alexandra Tietze, Sven Künzel, Sören Franzenburg, Camilo Barbosa, Gunther Jansen, Hinrich Schulenburg

**Affiliations:** 1 Evolutionary Ecology and Genetics, Department of Zoology, Christian-Albrechts-University of Kiel, Kiel, Germany; 2 Department of Evolutionary Genetics, Max-Planck-Institute for Evolutionary Biology, Plön, Germany; 3 Genetics and Bioinformatics, Department of Clinical Molecular Biology, Christian-Albrechts-University of Kiel, Kiel, Germany; 4 Division of Infectious Diseases, Department of Internal Medicine, University of Michigan, Ann Arbor, MI USA; 5 Antibiotic Resistance Group, Max-Planck-Institute for Evolutionary Biology, Plön, Germany

**Keywords:** Experimental evolution, Bacterial evolution

## Abstract

During antibiotic treatment, the evolution of bacterial pathogens is fundamentally affected by bottlenecks and varying selection levels imposed by the drugs. Bottlenecks—that is, reductions in bacterial population size—lead to an increased influence of random effects (genetic drift) during bacterial evolution, and varying antibiotic concentrations during treatment may favour distinct resistance variants. Both aspects influence the process of bacterial evolution during antibiotic therapy and thereby treatment outcome. Surprisingly, the joint influence of these interconnected factors on the evolution of antibiotic resistance remains largely unexplored. Here we combine evolution experiments with genomic and genetic analyses to demonstrate that bottleneck size and antibiotic-induced selection reproducibly impact the evolutionary path to resistance in pathogenic *Pseudomonas aeruginosa*, one of the most problematic opportunistic human pathogens. Resistance is favoured—expectedly—under high antibiotic selection and weak bottlenecks, but—unexpectedly—also under low antibiotic selection and severe bottlenecks. The latter is likely to result from a reduced probability of losing favourable variants through drift under weak selection. Moreover, the absence of high resistance under low selection and weak bottlenecks is caused by the spread of low-resistance variants with high competitive fitness under these conditions. We conclude that bottlenecks, in combination with drug-induced selection, are currently neglected key determinants of pathogen evolution and outcome of antibiotic treatment.

## Abstract (web summary)

Strong population bottlenecks in combination with weak antibiotic selection consistently favours the evolution of resistance across independently performed *Pseudomonas aeruginosa* evolution experiments.

## Main

Over the past decades, the spread of antibiotic resistance among pathogenic bacteria has become a global threat^1^. Because of comprehensive medical application and extensive use in agriculture, antibiotic resistance has increased steadily^2,3^. The contamination of natural environments with low antibiotic concentrations from wastewater might further contribute to resistance spread^4,5^. As a consequence, infections with antibiotic-resistant bacteria are predicted to be a major cause of death^6^ by 2050. New approaches in the fight against antibiotic resistance are thus desperately needed. Their successful development and implementation rely on in-depth understanding of the evolution of antibiotic resistance.

Evolution of drug resistance is not only influenced by the presence of the antibiotic per se. It is also affected by the drug concentration and the resulting selective constraints, as well as different population-genetic characteristics, such as population size and bottlenecks. Surprisingly, the combined influence of these factors has been largely neglected in current work on evolution of resistance, despite their likely relevance in vivo^7–9^. Populations of pathogenic bacteria undergo severe bottlenecks during an infection of a host, imposed by the host immune system, physical properties of infected tissues, transmission between hosts and, importantly, as a consequence of antibiotic treatment^10–13^, thereby linking population size and antibiotic-induced selection. In the presence of such bottlenecks, adaptation is strongly influenced by genetic drift, and the first beneficial variants that arise by chance usually face little clonal competition and have a higher probability of going to fixation^14,15^. Over time, this leads to periodic selection with decreased likelihood of parallel evolution^16–18^. By contrast, higher degrees of parallel evolution and clonal interference are expected in the case of weaker bottlenecks^14,15^. As higher genetic diversity is more likely to be maintained, the fittest variants tend to occur repeatedly under weak bottlenecks and steer the adaptative process^19–21^. In this context, variation in antibiotic dose alters the degree of selection on bacterial populations, often favouring different resistance mutations^22,23^. This is especially true in the case of trade-offs between resistance level or mechanism and competitiveness^7^.

The aim of our study is to assess to what extent bottleneck size and its likely interaction with antibiotic-induced selection affect evolution of drug resistance. To address this aim, we combined evolution experiments with whole-genome sequencing (WGS) and genetic analysis using *P. aeruginosa*, one of the three World Health Organization priority 1 most critical multidrug-resistant pathogens^24^. Experimental evolution was performed by serial dilution, for which we developed a protocol that achieves precise control of bottleneck size. *P. aeruginosa* reference strain PA14 was evolved over approximately 100 generations at two regular bottleneck sizes (BN): 50,000 or 5,000,000 cells, referred to as k50 or M5, respectively—and at three distinct selection levels (defined by the 0%, 20% or 80% inhibitory drug concentration: IC_0_, IC_20_ or IC_80_, respectively). Evolution experiments were performed for two antibiotics with different modes of action, the aminoglycoside gentamicin (GEN) or the fluoroquinolone ciprofloxacin (CIP). Genome sequencing and genetic analyses were used to identify the targets of selection and assess competitive fitness of the identified variants.

## Results

**Extended Data Fig. 1:**
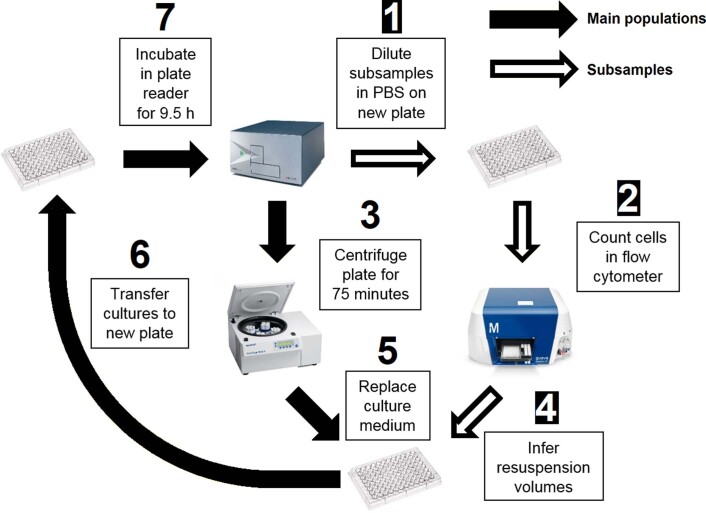
Design of evolution experiment. Numbers mark the succession of steps taken in the experimental protocol. Text in the boxes next to the numbers describe the experimental step. Arrows with black fill describe steps that are taken for the experimental cultures. Arrows with white fill describe steps that are taken for subsamples of the experimental cultures. The annotated steps were repeated for a total of 16 growth periods.

**Extended Data Fig. 2:**
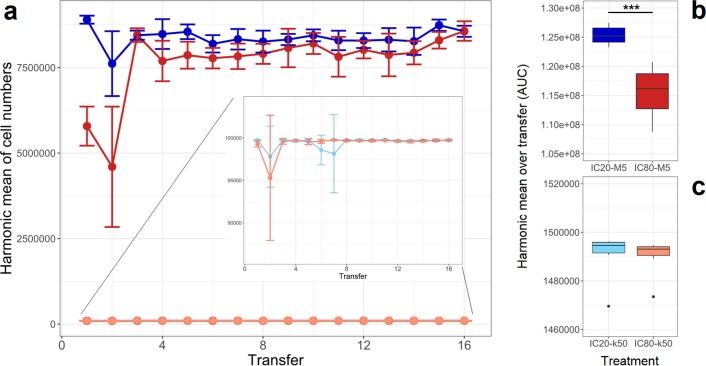
Harmonic means of cell concentrations during the GEN evolution experiment. **a**, Harmonic means per treatment group over time, **b**, AUC of harmonic means over transfer for weak bottleneck treatments, **c**, AUC of harmonic means over transfer for strong bottleneck treatments. The harmonic mean between starting population size and endpoint population size was calculated per growth period for every population. Panel a shows the mean and standard error for every group at every transfer. Panels b and c summarize the AUCs shown in panel a for every group. Blue: IC20 treatments; Red: IC80 treatments; light colours: k50 transfers; dark colours: M5 transfers. Significant difference between two treatment groups is indicated by *** (always p < 0.05 based on Welch t-test). Source data

**Extended Data Fig. 3:**
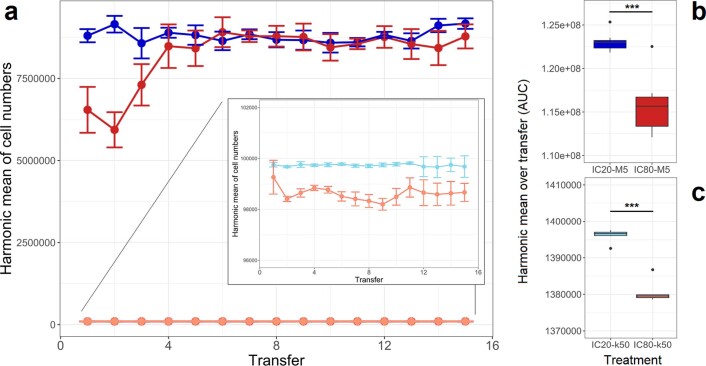
Harmonic means of cell concentrations during the CIP evolution experiment. **a**, Harmonic means per treatment group over time, **b**, AUC of harmonic means over transfer for weak bottleneck treatments, **c**, AUC of harmonic means over transfer for strong bottleneck treatments. The harmonic mean between starting population size and endpoint population size was calculated per transfer period for every population. Panel a shows the mean and standard error for every group at every transfer. Panels b and c summarize the AUCs shown in panel a for every group. Blue: IC20 treatments; Red: IC80 treatments; light colours represent k50 transfers; dark colours represent M5 transfers. Significant difference between two treatment groups is indicated by *** (always p < 0.05 based on Welch t-test). Source data

We performed fully independent experiments with the antibiotics GEN and CIP, but otherwise identical experimental design (Extended Data Fig. 1). On the basis of cell counts at the beginning and end of each growth period, we calculated the harmonic mean of population size and found that the experimentally controlled bottleneck size was indeed the main determinant of population-size differences among treatments (Extended Data Figs. 2 and 3 and Supplementary Table 1), thereby confirming the general suitability of our experimental design for assessing the influence of bottlenecks on evolution of resistance. Nevertheless, antibiotic concentration has an additional, smaller influence on this integrative measure of population size, especially at the beginning of the experiment (Extended Data Figs. 2 and 3). We then studied the evolutionary response to antibiotics for two types of traits—bacterial fitness and antibiotic resistance—each assessed using two approaches. As a proxy for bacterial fitness, we analysed the overall yield relative to the no-drug IC_0_ control, inferred from counts of viable cells at the end of each growth period across the evolution experiment, and growth rates, inferred from continuous optical density (OD) measurements throughout the evolution experiments (Methods). For resistance analysis, we used an integrative resistance estimate for bacterial populations from the end of the evolution experiment, measured as area under the curve (AUC) of a standardized dose–response curve, and proxies for minimum inhibitory concentrations (MIC), inferred from the standardized dose–response curves for the final populations (Methods).

**Fig. 1:**
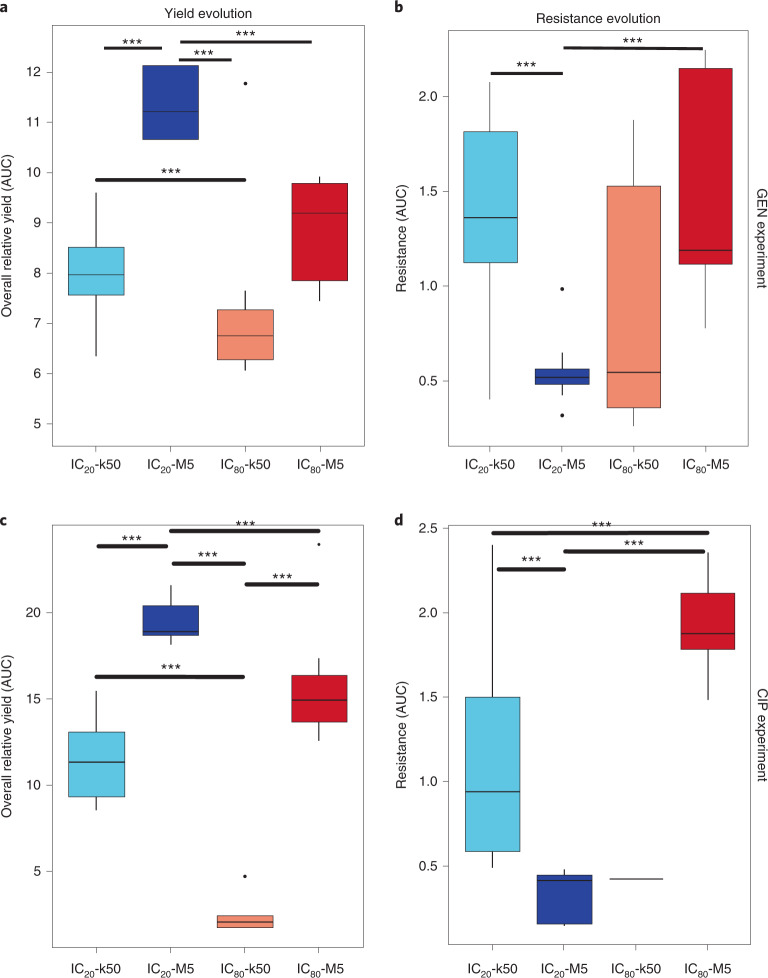
Variation in bottleneck size and drug-induced selection leads to consistent responses across two independent evolution experiments with distinct antibiotics. **a**,**b**, Results of the GEN experiment, showing changes in overall yield across experimental evolution relative to the no-drug control IC_0_ (**a**) and changes in overall resistance (**b**). **c**,**d**, Results of the CIP experiment, showing changes in overall yield across experimental evolution (**c**) and variation in resistance (**d**). The *x*-axis and colours represent different treatment groups (blue, IC_20_; red, IC_80_; light colours, 50k transfers; dark colours, 5 M transfers). For each replicate population, overall yield was inferred from counts of viable cells determined by flow cytometry at the end of each transfer period and related to the cell counts of the corresponding no-drug control of the same bottleneck size, followed by calculation of the AUC of yield across transfer periods of the evolution experiment. Resistance is shown as AUC of a standardized dose–response curve for the bacterial populations from the end of the evolution experiment (Methods and Extended Data Fig. 6). For the IC_80_-k50 treatment of the CIP experiment, only 1 out of 8 replicate populations survived, whereas in all other cases all 8 replicate populations per treatment were considered. In all box plots, which the centre line indicates the median, the box represents the data between first and third quantiles, the whiskers show the whole data range excluding outliers, and the dots indicate outliers (if present). Variation among treatments was evaluated with a general linear model; significant difference between two treatment groups, ***P < 0.05; Tukey’s honest significant difference (HSD) tests. Source data

**Extended Data Fig. 4:**
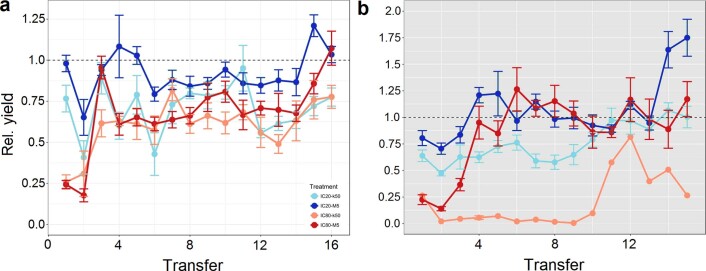
Evolutionary yield dynamics of the different treatment groups. **a**, Gentamicin experiment, **b**, Ciprofloxacin experiment. X-axes represent the time series of the evolution experiment: every point represents the end of a growth period before the next transfer. The Y-axes represent the mean yield of the treatments based on the cell counts obtained from flow cytometry. Error bars represent standard error of mean (8 replicates, 1 in CIP-IC80-k50). Blue: IC20 treatments; Red: IC80 treatments; light colours represent k50 transfers; dark colours represent M5 transfers. Source data

**Extended Data Fig. 5:**
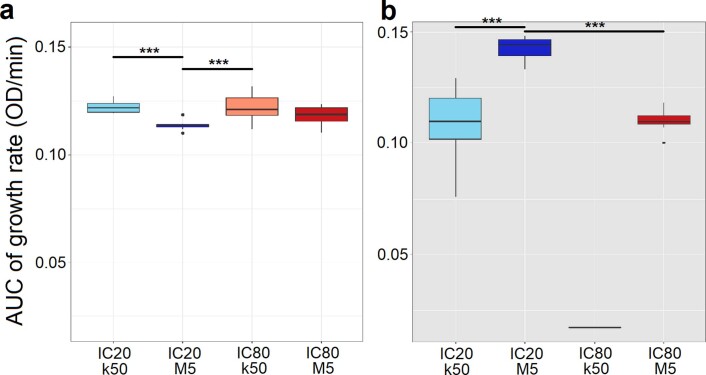
AUC of growth rates across evolution experiments. **a**, Gentamicin experiment, **b**, Ciprofloxacin experiment. X-axes represent the treatment groups. The Y-axes represent the AUC of growth rates, calculated for each growth period for every population. Error bars represent standard error of mean (8 replicates, 1 in CIP-IC80-k50). Blue: IC20 treatments; Red: IC80 treatments; light colours: k50 transfers; dark colours: M5 transfers. Significant difference between two treatment groups is indicated by *** (always p < 0.05 based on TukeyHSD tests). Source data

**Extended Data Fig. 6:**
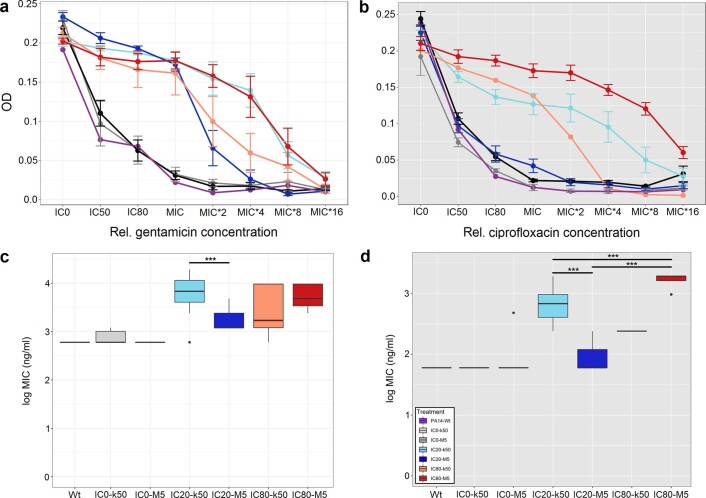
Dose response tests of evolved populations. a + c: Gentamicin experiment, b + d: Ciprofloxacin experiment. **a, b**, Dose Response Curves: X-axes represent the levels of tested drug concentrations relative to the PA14 reference. The Y-axes represent the final OD of the tested bacterial populations at 600 nm after 12 hours of incubation. Error bars represent standard error of mean (8 replicates). See Extended Data Table A E for summary of tested drug concentrations. **c, d**, Minimum inhibitory concentrations obtained from resistance assays: X-axes represent the treatment groups during the evolution experiments and the ancestral wildtype PA14. The Y-axes represent the lowest drug concentration of the resistance assays (ng/ml, logarithmic scale), for which growth was no longer detectable. Evolved populations were incubated for 12 hours at antibiotic concentrations summarized in Extended Data Table AE. Growth was determined by optical density measurements. Significant difference between two treatment groups is indicated by *** (always p < 0.05 based on TukeyHSD). Purple: PA14 ancestor; Grey: evolved no-drug controls; Blue: IC20 treatments; Red: IC80 treatments; light colours represent k50 transfers; dark colours represent M5 transfers. Source data

Across both evolution experiments, we consistently observed the highest overall yields for treatment with weak bottlenecks (that is, IC_20_-M5 and IC_80_-M5), followed by the IC_20_-k50 and IC_80_-k50 treatments (Fig. 1a,c, Extended Data Fig. 4 and Supplementary Tables 2 and 3). Our analysis of growth rates as an alternative proxy for fitness produced a consistent pattern for the CIP experiment (Extended Data Fig. 5 and Supplementary Table 4). However, for the GEN experiment, growth rates were significantly lower for the IC_20_-M5 treatment, while growth rates for the other treatments did not vary from each other (Extended Data Fig. 5 and Supplementary Table 4). In this case, the comparatively high yields achieved under IC_20_-M5 cannot rely on growth rate alone. We further consistently observed that the variation in overall yield only partially translated into final resistance. Irrespective of the drug and the approach used for resistance analysis, the highest resistance was always observed for the IC_20_-k50 and IC_80_-M5 treatments, in contrast to lower resistance in the IC_20_-M5 and IC_80_-k50 treatments (Fig. 1b,d, Extended Data Fig. 6 and Supplementary Tables 5–8). All antibiotic treatments produced higher resistance than the ancestor or no-drug IC_0_ control (Extended Data Fig. 6 and Supplementary Tables 5–8). Together, these data suggest that *P. aeruginosa* is better able to adapt under weak bottlenecks and produce higher bacterial yields, irrespective of the applied antibiotic. Interestingly, the ability to produce high yield is not necessarily the result of evolving higher levels of resistance, thus indicating that selection acts distinctly on these two parameters.

**Fig. 2:**
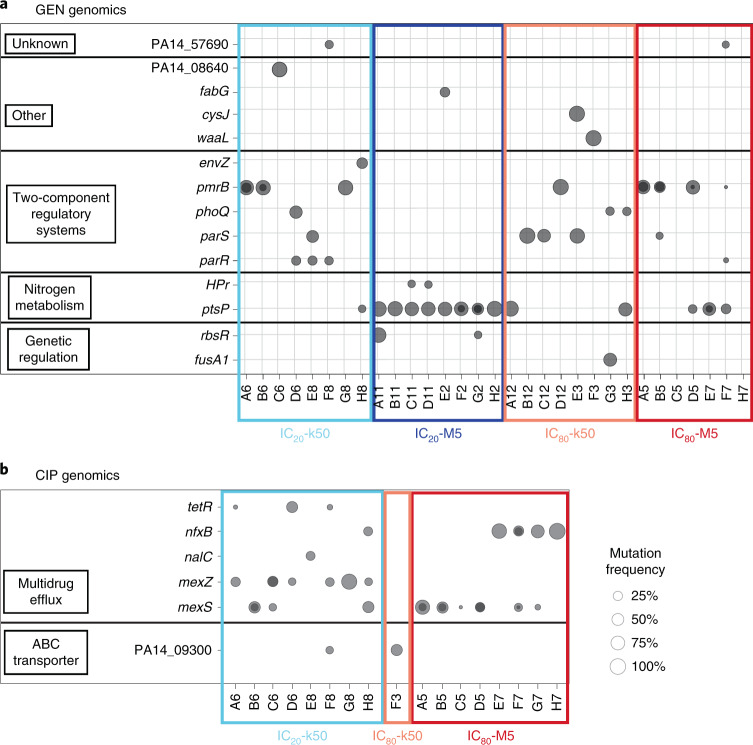
Weak bottlenecks increase the level of parallel genome evolution in the populations from the end of the evolution experiments. **a**, Results for the populations from the end of the evolution experiment with GEN, in which most mutations were found in two-component regulators and *ptsP*. All eight replicates were used for the different treatments, except for the IC_80_-M5 G7 population, which could not be recovered. **b**, Results for final populations from the CIP evolution experiment, which mainly harboured mutations in genes affecting multidrug efflux pumps. Results are shown for all eight replicate populations of the IC_20_-k50 and IC_80_-M5 treatments, and for the only surviving IC_80_-k50 population; the eight sequenced IC_20_-M5 populations did not harbour any sequence variants. The *x*-axes show the replicate populations and the coloured boxes show the treatments (light blue, IC_20_-k50; dark blue, IC_20_-M5; light red, IC_80_-k50; dark red, IC_80_-M5). The *y*-axes show mutated genes, sorted by their function, as indicated on the left. The size of the dots denotes the frequency of a particular mutation within a population. Dark dots indicate the presence of more than one mutation per gene. Source data

**Fig. 3:**
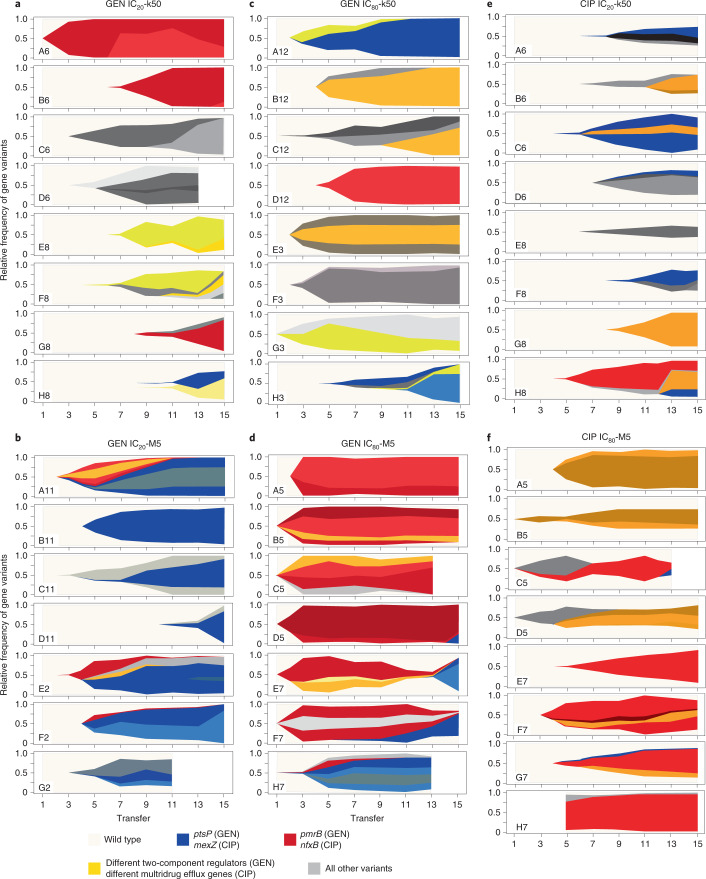
Weak bottlenecks generally lead to early spread of new variants and more competitive dynamics, but only few affected genes across replicate populations during the evolution experiments. **a**–**d**, Results for replicate populations from the GEN evolution experiment for IC_20_-k50 (**a**), IC_20_-M5 (**b**), IC_80_-k50 (**c**) and IC_80_-M5 (**d**). The IC_20_-M5 H2 population and the IC_80_-M5 G7 population were excluded because they could not be recovered from across the evolution experiment. **e**,**f**, Results for replicate populations from treatments of the CIP evolution experiment for IC_20_-k50 (**e**) and IC_80_-M5 (**f**). The *x*-axes represent the transfer period, the *y*-axes show the relative frequency of gene variants in a population. Population names are shown in the bottom left corner of each plot. The main colours denote the most frequently affected genes with variants for each of the two evolution experiments. Different shades further indicate different variants of the gene of that colour. Extended Data Figs. 7 and 8 show frequency dynamics of the individual mutations. Source data

**Extended Data Fig. 7:**
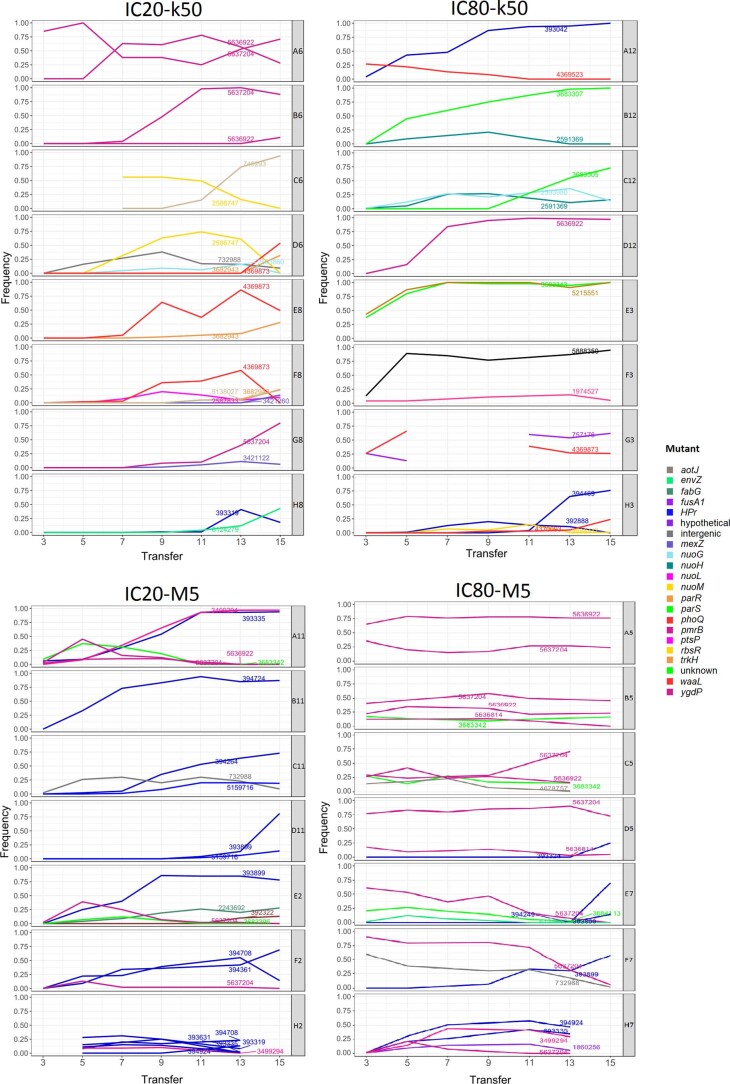
Allele dynamics of different treatment groups during the gentamicin evolution experiment. Group names are given on top of the graphs. Population names are given in the boxes right of the graphs. The X-axes represent the transfer period. The Y-axes represent the relative frequency of alleles in a population. Colours refer to different genes that the mutations appear in (see legend on right). Line annotations refer to the position of the mutation in the PA14 genome. Source data

**Extended Data Fig. 8:**
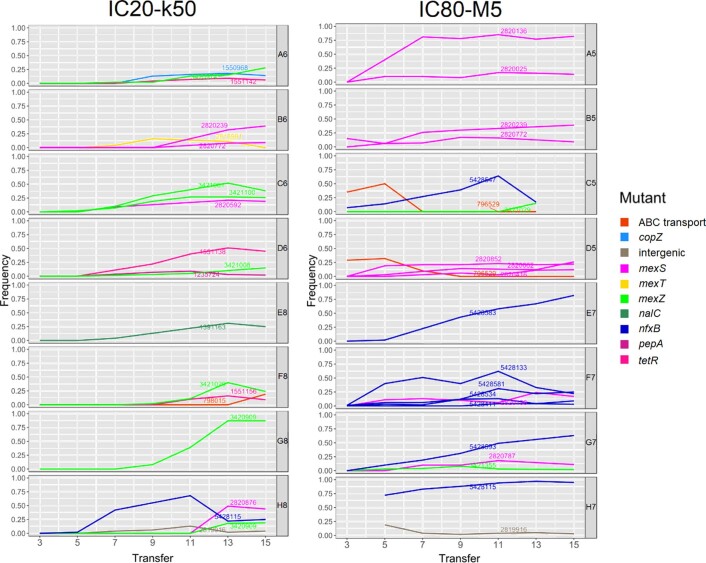
Allele dynamics of different treatment groups during the ciprofloxacin evolution experiment. Group names are given on top of the graphs. Population names are given in the boxes right of the graphs. The X-axes represent the transfer period. The Y-axes represent the relative frequency of alleles in a population. Colours refer to different genes that the mutations appear in (see legend on top right). Line annotations refer to the position of the mutation in the PA14 genome. Source data

**Extended Data Fig. 9:**
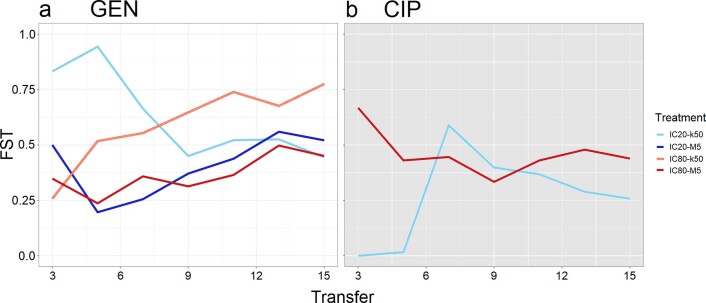
F_ST_ dynamics for mutated genes of all treatment groups during experimental evolution. **a**, F_ST_ dynamics of gentamicin evolution experiment, **b**, F_ST_ dynamics of ciprofloxacin evolution experiment. The X-axes represent the transfer period. The Y-axes represent the respective F_ST_ score. Colours refer to different treatment groups: Light blue: IC20-k50, Dark blue: IC20-M5, Light red: IC80-k50, Dark red: IC80-M5. Source data

Our subsequent genome analysis of the populations from the GEN experiment revealed that high population variant frequencies were primarily found in two genes (*ptsP* and *pmrB*) under weak bottlenecks, but in more genes under strong bottlenecks, and distinct variants emerged in environments with different antibiotic concentrations (Figs. 2 and 3 and Extended Data Figs. 7–9). In detail, we used WGS to identify the possible targets of selection and to characterize variant frequencies, either for the final populations from the evolution experiment (Fig. 2; total of 48 populations for the two evolution experiments) or across time (Fig. 3 and Extended Data Figs. 7 and 8; total of 46 populations, each assessed over 7 time points for the two experiments). For the GEN experiment, we found variants in more than a dozen genes with different functions. Among these, the most frequently mutated genes were the two-component regulatory systems PmrAB, ParRS and PhoPQ, as well as the gene *ptsP*, which is involved in nitrogen metabolism (Figs. 2a and 3). Weak bottlenecks led to high-frequency variants in only few genes: for IC_20_-M5, mainly in *ptsP*, and for IC_80_-M5, mainly in *ptsP* and *pmrB* (Supplementary Table 9). Interestingly, variants in *ptsP* emerged and reached detectable frequencies across all replicate populations more frequently than variants in other genes (Supplementary Table 9), possibly indicating a mutational bias. Nevertheless, this possible bias did not lead to predominance of *ptsP* variants across treatments.

In the GEN experiment, the divergence of favoured gene variants was higher in the populations that faced strong bottlenecks, both at the end of the evolution experiment and across time (Figs. 2a and 3 and Extended Data Fig. 7). This resulted in consistently more strongly differentiated replicate populations compared with the populations that experienced weak bottlenecks (indicated by higher *F*_ST_ values; Extended Data Fig. 9a). Moreover, variant frequencies increased more slowly when undergoing low-selection treatments rather than with high-selection treatments (IC_20_ compared with IC_80_ treatments; Fig. 3). Particularly for the low-selection and strong-bottleneck treatment IC_20_-k50, new variants usually did not reach detectable frequencies before transfer period 7 or 9, and high variant frequencies developed only during the second half of the experiment (Fig. 3a). In contrast, the IC_80_-M5 treatment usually showed high frequencies of new favoured variants by transfer 3 (Fig. 3d). In general, more competitive dynamics, consisting of more simultaneous variants at particular time points, appear to be common in treatments with weak bottlenecks (5 out of 7 replication populations each for the IC_20_-M5 and IC_80_-M5 treatments; Fig. 3c,d).

In the CIP experiment, only two treatment groups yielded information on variant distribution. For these treatments, the genomic results appear to be consistent with the GEN experiment. Only one population survived the high-selection and strong-bottleneck treatment IC_80_-k50, thus precluding more detailed genome comparisons. For the remaining two treatments, we found variants in six genes, almost all affecting multidrug efflux (Fig. 2b). The different CIP treatment groups appeared to favour variants in different genes: *mexZ* variants occurred exclusively under IC_20_-k50, whereas *mexS* and *nfxB* variants occurred primarily under IC_80_-M5 conditions (Supplementary Table 10). Moreover, weak-bottleneck treatments appeared to produce variants in a smaller number of genes (Fig. 2b), which then also reached high frequencies much earlier than in strong-bottleneck treatments (Fig. 3e,f and Extended Data Fig. 8), resulting in lower *F*_ST_ indices for population differentiation (Extended Data Fig. 9b) and low diversity indices (Supplementary Table 11). The most important difference between the CIP and GEN experiments is that we could not detect any new variants for the low-selection and weak-bottleneck treatment IC_20_-M5. Since identical methods were used for WGS analysis across treatment groups, the genomic response of this treatment is clearly distinct from those of the other treatments. This result suggests that the observed response in yield under IC_20_-M5 conditions (Fig. 1c) results from phenotypic, non-heritable adaptations.

**Fig. 4:**
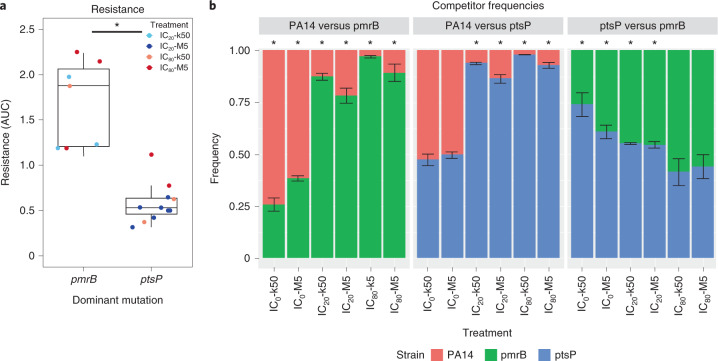
*ptsP* variants confer higher competitiveness under low GEN concentrations yet lower GEN resistance than *pmrB* variants. **a**, Populations with the most frequent mutation in *ptsP* showed significantly lower resistance than populations with the most frequent mutation in *pmrB*. The *x*-axis represents genes affected by most frequent mutations. The *y*-axis represents AUC as a proxy for resistance to GEN. *P < 0.05, general linear model (GLM) statistical analysis. **b**, *ptsP* variants had a competitive advantage over *pmrB* variants in competition experiments with low drug concentrations. The *x*-axis represents the different treatment conditions. The *y*-axis shows the relative frequency of the competitors. Colours indicate competitor variant or wild type. In competition experiments, cultures of competing strains were mixed at a 1:1 ratio. Data are mean ± s.e.m. of three independent biological replicates for each treatment. The mean of each biological replicate was based on five technical replicates (Extended Data Fig. 10). Asterisks at the top indicate significant difference in frequency between the two competitors (*P < 0.05; two-sample *t*-test). Source data

**Extended Data Fig. 10:**
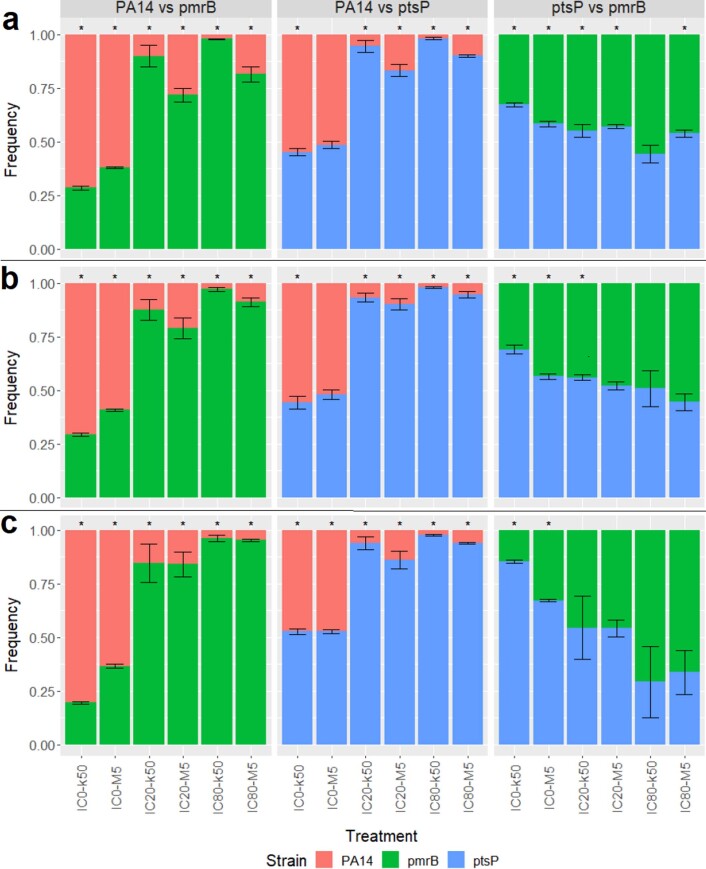
Competition outcomes of the three separate experiments. **a**, A12 vs D12. Clones originated from the IC80-k50 evolution treatment. **b**, A6 vs B11. Clones originated from the IC20 evolution treatments. **c**, A5 vs E7. Clones originated from the IC80-M5 evolution treatment. Different mutant clones, which evolved in the GEN evolution experiment, competed against one another. Competitions included eight technical replicates for every condition. The X-axis indicate the different competition treatment conditions. The Y-axes represent the relative frequency of the clones. Colours refer to different clonal variants: Red = Wt, Green = pmrB, Blue = ptsP. Error bars represent the standard error based on five technical replicates for each treatment. Asterisks represent significant difference in frequency between two strains (* p < 0.05; two sample t-test). Source data

While *ptsP* variants emerged and reached detectable frequencies more often than variants in other genes across the entire GEN evolution experiment (Supplementary Table 9), they did not predominate in all treatment groups. For example, *pmrB* variants appear to be at least as strongly favoured as the *ptsP* variants under IC_80_-M5 conditions, especially if temporal dynamics are considered (Figs. 2a and 3a–d). To further assess the differential success of *ptsP* and *pmrB* variants, we specifically characterized variants of these genes and found opposite effects in resistance and competitive fitness (Fig. 4). First, we used a subset of the obtained GEN-resistance data (see Fig. 1b) to assess whether populations with high frequencies of either *pmrB* or *ptsP* variants differ in resistance, revealing that variants in *pmrB* are significantly more resistant than variants in *ptsP* (Fig. 4a and Supplementary Table 12). This suggests that the *pmrB* variants should have been selectively favoured in all treatments with antibiotics, which however was not the case (Figs. 2 and 3). We then assessed the competitive fitness of individual variants at different antibiotic concentrations and bottleneck sizes. For this, we isolated three strains with a single variant in *ptsP* and three strains with a single variant in *pmrB* from different evolved populations of the same or comparable evolution treatments (in all cases, the isolated variants showed high frequencies in the genomic analysis; Supplementary Table 13). Additional WGS revealed that the isolated strains only varied from the ancestor at the expected mutations in either *ptsP* or *pmrB* (Methods). These strains with either *ptsP* or *pmrB* variants were each combined with either the ancestral wild-type PA14 reference strain or with each other, always in a 1:1 competition ratio and for a single growth period of 12 h, followed by variant frequency analysis using amplicon sequencing. We found that the *ptsP* variants possessed significantly higher competitive fitness than *pmrB* variants under low antibiotic concentrations or absence of drugs (IC_0_ and IC_20_ treatments; Fig. 4b, Extended Data Fig. 10 and Supplementary Table 14). However, no significant difference in *pmrB* variant frequencies versus *ptsP* was detected under IC_80_ conditions. In summary, *ptsP* variant frequencies decreased with increasing antibiotic concentration. The competition against PA14 in drug-free environments showed an equal frequency of *ptsP* and a lower frequency of *pmrB* variants compared with the ancestor. All resistant strains outcompeted PA14 in the treatment groups with antibiotics (Fig. 4b, Extended Data Fig. 10 and Supplementary Table 14).

## Discussion

Bottlenecks occur frequently in natural populations of pathogenic bacteria and have a critical role in pathogen adaptation during infection and transmission^25–27^. The interplay of bottlenecks and antibiotic-induced selection is therefore likely to be key for evolution of resistance in vivo, but is widely neglected in the current literature on antibiotic resistance. Here, using an approach in which population bottleneck size is controlled by counting cells with flow cytometry, we demonstrate a consistent and reproducible interplay of the two factors on the dynamics of resistance evolution in two independent sets of evolution experiments. By varying both factors, we could specifically assess which aspects of bacterial adaptation are affected by a strong bottleneck alone. As predicted, we found that high resistance emerged when bottlenecks were weak and selection was strong. Surprisingly, however, high resistance also repeatedly evolved with strong bottlenecks and weak selection, most probably because the lower selective constraints reduced the bottleneck-induced probability of losing favourable alleles. Moreover, in one of the experiments, weak bottlenecks and weak selection favoured the spread of variants with low-level antibiotic resistance but higher competitive fitness than more resistant variants, leading to a low degree of resistance under these conditions. Furthermore, strong bottlenecks increased variation across replicate populations, most probably due to high genetic drift, resulting in little parallel evolution. Together, our results provide evidence for a critical influence of both bottlenecks and the level of antibiotic-induced selection on the dynamics of evolution of antibiotic resistance.

Our main findings were consistent across the two independent sets of evolution experiments, which were performed with distinct antibiotics from two different classes, aminoglycosides and fluoroquinolones, and with several independent replicates per treatment. Across these two sets of experiments, we found that the larger starting population size under weak bottlenecks often favoured competition between independent mutations, with one ultimately being lost, depending on the strength of antibiotic selection (Figs. 2 and 3). This observation agrees with the original expectation that weak bottlenecks facilitate a higher degree of parallel evolution because of the increased probability of the fittest mutants occurring and outcompeting others^24^. In accordance with this expectation, populations experiencing weak bottlenecks had a comparatively high overall yield (Fig. 1a,c), indicating that the bacteria were indeed able to respond to the respective selection conditions. By contrast, strong bottlenecks in both sets of evolution experiments led reproducibly to a comparatively late spread of variants and large variation among replicate populations in the variant genes, highlighting the strong influence of genetic drift under these conditions (Figs. 2 and 3).

There are two important exceptions to the above patterns. First, in the CIP experiment, we could not detect any variants spreading under weak bottlenecks combined with low selection levels (that is, the IC_20_-M5 treatment), even though populations increased their overall yield (Fig. 1c). This result may suggest that the evolutionary response is due to genomic rearrangements, which cannot be easily inferred from the short-read sequencing data obtained for this study. As an alternative explanation, phenotypic responses are sufficient to counter the low selective constraints imposed, thus allowing the wild-type bacteria to proliferate (Fig. 1c) and to outcompete any low-resistance variants, which were observed to spread in the GEN experiment under the IC_20_-M5 conditions (Figs. 1a, 2a, 3b and 4). Second, almost all populations went extinct when experiencing strong-bottleneck and high-selection conditions in the CIP experiments. This result indicates that this combination of conditions imposed a double constraint on the bacterial populations: the high inhibitory concentration of the drug continued to constrain population size after the bottleneck—as confirmed by analysis of an integrative measure of population size across one growth period, especially at the beginning of the evolution experiments (Extended Data Figs. 2 and 3)—thus decreasing the probability of favourable mutations to arise and reach sufficiently high frequencies. As the latter effects were particular to the experiments with CIP, the exact causes underlying the generally consistent overall results seen in Fig. 1 may vary, depending on the antibiotic and possibly the probability of resistance mutations arising de novo.

Surprisingly, populations consistently evolved a high yield and high resistance under IC_20_-k50 conditions, especially compared with the treatment with a strong bottleneck (IC_80_-k50; Fig. 1). We reason that this is probably because the lower drug concentration allows the populations to recover and maintain comparatively higher population size than the corresponding higher-drug concentration condition, as confirmed for the beginning of the evolution experiments by our analysis of the integrative measure of population size (Extended Data Figs. 2 and 3). During such a recovery phase, a newly arisen resistant variant that survived drift during the bottleneck can spread and reach sufficiently high frequencies to be maintained over longer time periods or even become fixed in the population. Moreover, the larger population size during the recovery phase increases the probability of favourable mutations occurring, further enhancing the ability of the bacterial populations to adapt. Both processes are less likely under the IC_80_-k50 conditions, where strong bottlenecks combined with high selection levels constrain population size over longer time periods (Extended Data Fig. 3 and 4).

Unexpectedly, high overall yield in the weak-bottleneck treatments did not directly translate to high antibiotic resistance (Fig. 1), especially in the GEN evolution experiments, for which this treatment consistently resulted in evolutionary changes (confirmed by the observed genetic changes; Figs. 2 and 3). Moreover, in the IC_20_-M5 treatment of the GEN experiment, high overall yield coincided with low growth rates, suggesting that the high cell counts were not caused by the rate of replication alone and there was not a simple linear relationship between these two components of bacterial fitness (that is, reproductive rate and final population size). For a further evaluation of this finding, we performed competition experiments among variants in two genes, *ptsP* and *pmrB*, which were most commonly affected in either IC_20_-M5 or IC_80_-M5 treatments. These experiments (Fig. 4b) demonstrate that the variations in resistance levels and competitive fitness are jointly responsible for the evolutionary success of the variants across treatments. PmrB is a sensory kinase of the two-component regulatory system PmrAB, which regulates lipopolysaccharide modifications of the bacterial outer cell membrane, specifically lipid A^28–30^. Lipid A modification is often found in aminoglycoside-resistant bacteria^31^. PtsP is a phosphoenolpyruvate-dependent phosphotransferase that transfers the phosphoryl group from phosphoenolpyruvate to the phosphoryl carrier protein (NPr)^32^. It has an important role in the nitrogen cycle and glucose transport in bacteria^32,33^. However, the role of *ptsP* in aminoglycoside resistance is poorly understood^34^. Deletions of *ptsP* have been shown to cause overproduction of pyocyanin, a toxin produced by *P. aeruginosa* that oxidizes other molecules^35^. Moreover, *ptsP* mutations can increase resistance to antimicrobial peptides and expression of the major quorum-sensing regulators *lasI* and *rhlI*^35,36^. Our work highlights that different variants in *ptsP* can also confer resistance against the aminoglycoside GEN, albeit at a lower level than mutations in *pmrB*. Importantly, the lower resistance levels of the *ptsP* variants were associated with a higher competitive fitness than the *pmrB* variants under low antibiotic concentrations, explaining their predominance under these conditions. Interestingly, *ptsP* and *pmrB* variants had similar competitive fitness under high antibiotic dose, resulting in variants of either gene going to fixation under these conditions (Figs. 2a and 3d).

Our study expands the insights obtained from the few previous experiments on the influence of bottlenecks on the evolution of antibiotic resistance. For example, a recent study assessed evolution of *Escherichia coli* under continuously increasing concentrations of CIP and with three bottleneck sizes, two in the range of the inverse of the frequency of drug target mutations (that is, 10^8^ and 10^10^ cells), and a third with a single-cell bottleneck^12^. Even though the exact experimental design was different, the results similarly highlighted that variation in bottleneck size impacts evolutionary trajectories and that weak bottlenecks generally lead to high resistance and a high degree of parallel evolution. Interestingly, the largest bottleneck size favoured the spread of variants in drug target genes, especially *gyrA*, with apparently no fitness costs, whereas the intermediate bottleneck size led to selection of different gene variants, all with apparently large fitness costs^12^. By contrast, we did not identify mutations in the drug target genes and no substantial differences in growth costs under drug-free conditions for the high-resistance variants. A possible reason for the absence of drug target gene variants, which are often found in clinical *P. aeruginosa* isolates from patients treated with aminoglycosides and fluoroquinolones (for example, in *fusA1* or *gyrA*)^37,38^, is that we used antibiotics at sub-MIC levels, to enable populations to survive initially and adapt to the imposed treatments. A second example previously assessed adaptation of *Pseudomonas fluorescens* to rifampicin under three bottleneck sizes, similarly revealing an impact of bottleneck size on the paths of resistance evolution^39^. In particular, variants in the main resistance gene *rpoB* spread under both strong and weak bottlenecks, yielding high levels of resistance and a high degree of parallel evolution under these conditions. By contrast, intermediate bottleneck sizes favoured mutations in a variety of genes, including a regulatory gene, yielding little parallel evolution. This study applied relative bottleneck sizes (rather than absolute bottleneck sizes, as in our study) and used a higher carrying capacity. As a consequence, the number of generations over the course of a growth period, and thus the likelihood of emergence and spread of favourable resistance mutations, is increased^15,40^, leading to the observed high resistance and parallel evolution even under strong bottlenecks. None of the previous studies assessed an interplay of bottlenecks and antibiotic-induced selection. As antibiotics themselves affect population size, similar to other environmental stressors, the adaptive consequences of a bottleneck need to be related to the relevant selective constraint that challenges the evolving populations. By experimentally controlling the interaction of both factors, our study allows us to increase the understanding of how populations adapt to stressful environments under different bottleneck intensities.

Bottlenecks are a characteristic property of the infection process^10,11,25,27,41–44^. Thus, knowledge of their impact on adaptation is essential for a full understanding of pathogen evolution and for the design of effective therapy. Our study provides a proof of concept that the interplay of bottleneck size and antibiotic-induced selection consistently alters the evolutionary trajectories to resistance and competitive fitness of adapting bacteria. Strong bottlenecks, which are likely to be prevalent in vivo, lead to more diverse outcomes under both high and low antibiotic concentrations, making predictions on evolution of resistance and the prevailing resistance variants exceedingly difficult. This has important consequences for the optimization of treatment design, which is unlikely to be universally applicable for a specific type of infection. Instead, treatment would have to be adapted on an individual patient level, taking into account the selected resistance mechanism(s) in the pathogen population and, importantly, the associated fitness costs^45^.

## Methods

### Materials

All experiments were performed with *P. aeruginosa* PA14^46^ and mutants thereof, which emerged during the evolution experiments. Bacteria were grown in M9 minimal medium, consisting of 7 g l^−1^ K_2_HPO_4_, 2 g l^−1^ KH_2_PO_4_, 0.588 g l^−1^ Na_3_C_6_H_5_O_7_, 1 g l^−1^ (NH_4_)_2_SO_4_, 0.1 g l^−1^ MgSO_4_, and supplemented with 2 g l^−1^ glucose and 1 g l^−1^ casamino acids. Different single colonies from M9 agar plates (M9 supplemented with 15 g l^−1^ agar) were picked to initiate the independent replicate populations of the evolution experiments. Exponential phase cultures with an OD of 0.1 (equivalent to 10^4^–10^5^ colony-forming units per ml) were used as inoculum for resistance assays. For long-time storage, bacterial cultures were supplemented with 30% glycerol and frozen at −80 °C.

### Flow cytometry

A Guava easyCyte flow cytometer was used to assess cell counts of bacterial cultures during the evolution and competition experiments. Cells were suspended at an appropriate concentration of <1,500 cells per µl in PBS and analysed by flow cytometry using a flow rate of 0.236 µl s^−1^ for either 30 s or until a total cell count of 5,000 cells per sample was reached. Propidium iodide (1.9 mM) was used to stain dead cells and thus determine the number of viable cells during flow cytometry. For each culture, the total number of cells was calculated from the measured cell concentration, adjusted by the number of dead cells, sampling volume and dilution factor. These calculations were used to determine the transfer volume during the evolution experiment and to infer final yield at the end of each season as a proxy for bacterial fitness.

### Dose–response curves and drug concentration determination for the ancestral populations

We used dose–response curves in broth cultures and OD measurements as a proxy for bacterial growth, consistent with standard diagnostic approaches for measurement of antibiotic resistance (for example, the Vitek 2 approach (bioMérieux)). Bacterial growth was assessed in 100 µl volumes at ten different antibiotic concentrations in a 96-well plate using a fully randomized design, including eight technical replicates per concentration, eight no-drug controls and eight medium-only controls. Growth was measured as OD after 12 h at 37 °C under constant shaking (double-orbital, 900 rpm) in Tecan plate readers. The lowest antibiotic concentration for which no visible growth was optically measurable was taken as the drug’s MIC.

### Design and general setup of evolution experiments

We developed a protocol for experimental evolution that includes cell counting by flow cytometry to ensure that an exact number of cells are transferred across growth periods (Extended Data Fig. 1). The evolution experiment was run in 96-well plates over 16 growth periods and included two distinct transfer bottleneck sizes (BN of 50,000 cells and 5,000,000 cells) and three inhibitory antibiotic concentrations (IC_0_, IC_20_ and IC_80_), combined in a full factorial design. The evolution treatments were randomized across the 96-well plates using a block design and each included eight fully independent replicates. Experimental evolution was performed independently with two distinct antibiotics, the aminoglycoside GEN and the fluoroquinolone CIP.

In detail, after each growth period, a 4 µl subsample of each bacterial culture was diluted 1:1,000 in PBS in two steps in a new 96-well plate and then used to determine the exact cell count by flow cytometry. In parallel, the remainder of the bacterial culture was centrifuged at 5,000 rpm for 75 min to remove the old growth medium and resuspended in fresh M9 medium. The resuspension volumes were calculated individually, on the basis of the flow cytometry results. Resuspension volumes were set to achieve a concentration of 5 × 10^5^ cells per μl per culture. The plate for the next growth period was prepared accordingly (100 μl per well minus the calculated transfer volume) and the respective volumes were then transferred to the new plate: 0.1 μl for 50,000 cells and 10 μl for 5,000,000 cells. The freshly inoculated plate was sealed with transparent foil and incubated for 9.5 h in a Tecan plate reader at 37 °C under constant shaking (double-orbital, 900 rpm). OD measurements were taken at 15 min intervals throughout each growth period of the evolution experiments and later used for inference of growth rates. After every second transfer, a subsample of bacteria from the evolution experiments were supplemented with 30% glycerol and frozen at −80 °C for later use.

As the evolutionary potential of the bacteria is influenced by population size across entire growth periods and not only by the bottleneck size, we additionally assessed variation among treatments for an integrative measure of population size across entire growth periods. For this assessment, we calculated the harmonic mean of population size, using the cell numbers at the beginning of a growth period, which we controlled experimentally through our protocol (either 50,000 or 5,000,000) and the end-point population size, which we determined through flow cytometry, as described above. The AUC of harmonic mean population size was calculated and statistically compared with a general linear mixed model and Tukey’s HSD post hoc tests.

### Assessment of bacterial fitness

We calculated two proxies of bacterial fitness during the evolution experiment. First, we used the absolute cell counts, determined by flow cytometry at the end of each growth period, to infer final yield for each growth period and replicate population, followed by calculation of relative yield by dividing the cell counts for each replicate population with that for the corresponding no-drug control (either IC_0_-k50 for strong-bottleneck treatments or IC_0_-M5 for weak-bottleneck treatments). Relative yield was further summarized by calculating the AUC of yields across all growth periods of the evolution experiment. As final yield was based on absolute cell counts, we consider this measure an informative proxy for bacterial fitness, which reflects the number of cells achieved by each population under the various treatment conditions. Second, we used continuous OD measurements to calculate growth rate for each growth period and each replicate population using GrowthRate software^47^. Although these calculations become less reliable if growth rates are generally low, as in some treatment groups, we similarly considered growth rate to be an informative proxy for bacterial fitness. Growth rate was also summarized by calculating AUC across growth periods of the evolution experiment. For both measures, we statistically compared treatment groups with a general linear mixed model and Tukey’s HSD post hoc tests.

### Antibiotic resistance of evolved populations

Populations of the last transfer period were challenged with different concentrations of the treatment drug, to obtain dose–response curves for the evolved populations. For this assessment, all cultures were standardized to the same population size of 500,000 cells. All bacterial populations were consistently subjected to eight distinct antibiotic concentrations: two below (IC_50_ and IC_80_) and six above (2 × MIC, 4 × MIC, 8 × MIC and 16 × MIC) the MIC of the ancestral population. We specifically chose this design to ensure comparability of the experimental procedures for the evolved populations. Control measurements were performed in the absence of antibiotics. Kinetic OD measurements were performed in Tecan plate readers at 15 min intervals. As an integrative measure of antibiotic resistance, we used the end-point ODs of all concentrations to calculate the AUC of resistance for each replicate population. As an alternative measure of resistance changes, we also determined MIC as the lowest concentration for which the measured OD was below 0.01. If growth was still observed for the highest antibiotic concentration that was included in the experiment, then the MIC was set at this concentration, thereby yielding a conservative estimate for resistance increases in this case. For each of the two measures of resistance, we statistically compared variation among treatments using a general linear model and Tukey’s HSD post hoc tests.

### DNA sequencing and genomics

We performed WGS of entire populations, because this allows us to describe the overall pattern of allele frequency changes within the populations and thus the overall evolutionary response^48–50^. DNA was isolated from experimentally evolved populations and the original starting cultures, using an established cetyl trimethylammonium bromide-based extraction protocol^51^. WGS of DNA samples of the last transfer period was performed at the Competence Centre for Genomic Analysis. Material for two populations from the GEN treatment could not be recovered and was thus excluded from analysis. Sequencing libraries were generated with the Nextera DNA Flex library preparation kit and sequencing was performed on the Illumina HiSeq 4000 platform using the Illumina paired-end technology with read lengths of 150 bp and an average base coverage of > 100 (ref. ^52^). In addition, DNA extracted from transfers 3, 5, 7, 9, 11 and 13 were sequenced on the Illumina NextSeq platform at the Max Planck Institute for Evolutionary Biology (MPI-EB), with read lengths of 150 bp and an average base coverage of >40. Sequence reads were provided in the fastq format^53^. We excluded populations IC_20_-M5 H2 and IC_80_-M5 G7 from the GEN experiment, because material from some transfers could not be recovered. Quality and quantity of reads were checked with FastQC^54^. Trimmomatic was used to remove sequencing adapters from the Nextera library and to filter out low-quality reads^55^. High-quality reads were mapped to the UCBPP_PA14 reference genome with the software bwa^46,56^. The generated.bam files were scanned for SNPs, insertions and deletions using the variant calling programs FreeBayes, PinDel and VarScan^57–59^. The resulting output files were filtered for duplicates, ancestral variants and variants found in the evolved controls using R and additionally checked by visually inspecting the called genome positions provided by the.bam file in the IGV genome browser^60^. The detected variants were annotated with the help of SnpEff^61^ and the *Pseudomonas* database (available at http://pseudomonas.com). The R package ggmuller was used to generate Muller plots of the evolving populations. SNP frequencies were used to calculate *F*_ST_ at every transfer for all treatment groups in which high-frequency variants occurred. Haplotype diversity was calculated as

$$H = 1 - \mathop {\sum}\limits_{i = 1}^j {P_i}^2$$

where *P*_*i*_ represents the fraction of a haplotype in the population and *j* represents the total number of haplotypes. Haplotype diversity was calculated within individual populations of the treatment group (HS) and between the different replicates of the treatment group (HT). *F*_ST_ was then calculated as:

$$F_{\mathrm{ST}} = \frac{{{\mathrm{HT}} - \overline {{\mathrm{HS}}} }}{{{\mathrm{HT}}}}$$

We further performed WGS on the Illumina NextSeq platform of the MPI-EB as above to confirm that the isolated clones used in the competition experiments contained only a single variant, as expected from the genome data of the evolved populations.

### Competition assays and clone frequency analysis using amplicon sequencing

In total, three pairs of different *pmrB* and *ptsP* mutants were competed against one another and against the PA14 reference in 96-well plates (Supplementary Table 13). As a control, each single strain was incubated in individual wells under the same conditions on a separate 96-well plate. The clones were each picked as a single colony-forming unit from M9 agar plates, which had been inoculated with a cryo-preserved culture of an evolved bacterial population, which should contain only a single variant according to the performed WGS of bacterial populations. The picked clones were cultivated in 5 ml M9 medium at 37 °C. Cultures of competing strains were set to an OD of 0.1 mixed at a 1:1 ratio before inoculation of the competition cultures. The cultures were then transferred to 96-well plates. Competitions ran with culture volumes of 100 µl at different GEN concentrations for 12 h at 37 °C in a Tecan plate reader.

The relative frequency of the competing strains was determined using amplification of diagnostic genome regions and sequencing. Bacterial pellets from the end of the competition experiments were resuspended in 50 μl nuclease-free water and boiled for 15 min. A two-step PCR was subsequently performed to amplify the region of interest and to ligate barcodes to the amplicons. In detail, 1 μl of each lysate was used as a template for PCR amplification of the diagnostic loci (primer sequences are shown in Supplementary Table 15). One microlitre of each PCR product served as template for the second PCR to attach the individually barcoded sequencing primers (Supplementary Table 16). Both rounds of PCR were run for 15 cycles. DNA concentrations of every final sample were set to 100 ng μl^−1^ and 5 μl per sample was pooled in a single Eppendorf tube. The library mix was then further purified by gel extraction with the GeneJET gel extraction kit (Thermo Fisher Scientific). Sequencing was performed on the Illumina NextSeq platform of the MPI-EB. Reads were quality-filtered with Trimmomatic 0.39 and subsequently aligned to the PA14 reference genome by using bwa^46,55,56^. SAMtools was used to generate bam files that were evaluated with the Integrative Genomics Viewer^60,62^. The strain frequencies were calculated from the SNP counts. The mean frequencies of every biological replicate were calculated and then used to compare the frequencies between the three main variants (*ptsP*, *pmrB* and wild type) in competition with a two-sample *t*-test (*n* = 3).

### Reporting Summary

Further information on research design is available in the Nature Research Reporting Summary linked to this article.

## Supplementary information

Supplementary Information Supplementary Tables 1–16.

Reporting Summary

Peer Review Information

## Data Availability

WGS data for bacterial populations are available from GenBank at the National Center for Biotechnology Information under accession numbers PRJNA725112 and PRJNA725351. The experimental data are available from Dryad (10.5061/dryad.dncjsxm06). Source data are provided with this paper.

## Source data

Source Data Fig. 1 Statistical source data: relative yield during evolution and AUC of resistance.
Source Data Fig. 2 Statistical source data: Presence and frequency of variants after evolution.
Source Data Fig. 3 Statistical source data: Variant frequency dynamics during evolution.
Source Data Fig. 4 Statistical source data: Resistance of ptsP and pmrB variants and competitive frequencies.
Source Data Extended Data Fig. 2 Statistical source data: Harmonic mean of population size during evolution (GEN experiment).
Source Data Extended Data Fig. 3 Statistical source data: Harmonic mean of population size during evolution (CIP experiment).
Source Data Extended Data Fig. 4 Statistical source data: Relative yield dynamics during evolution.
Source Data Extended Data Fig. 5 Statistical source data: Growth rate during evolution.
Source Data Extended Data Fig. 6 Statistical source data: Dose response curves and relative MICs after evolution.
Source Data Extended Data Fig. 7 Statistical source data: Variant frequencies during evolution (GEN experiment).
Source Data Extended Data Fig. 8 Statistical source data: Variant frequencies during evolution (CIP experiment).
Source Data Extended Data Fig. 9 Statistical source data: Fixation index (*F*_ST_) dynamics during evolution.
Source Data Extended Data Fig. 10 Statistical source data: Competitive frequencies of all competition experiments.
