# Supplementary material for: Bottleneck size and selection level reproducibly impact evolution of antibiotic resistance
Source: Nat Ecol Evol. 2021 Jul 26;5(9):1233–42. doi: 10.1038/s41559-021-01511-2 (PMC8390372; doi:10.1038/s41559-021-01511-2)
Supplement: Supplementary file 1 — Supplementary Tables 1–16. [file 41559_2021_1511_MOESM1_ESM.pdf]

nature portfolio

https://doi.org/10.1038/s41559-021-01511-2

# Supplementary information

# Bottleneck size and selection level reproducibly impact evolution of antibiotic resistance

In the format provided by the authors and unedited

**Supplementary Table 1: Statistical results of Welch t-tests for area under curve (AUC) of harmonic mean Population sizes from the GEN and CIP experiments.**

| | GEN experiment | | | CIP experiment | | |
|---|---|---|---|---|---|---|
| **Comparison** | **t** | **Df** | **P** | **t** | **Df** | **P** |
| IC80 k50-IC20 k50 | 0.2012 | 13.279 | 0.8436 | 14.654 | 11.373 | **<0.0001** |
| IC80 M5-IC20 M5 | 5.6754 | 7.4104 | **0.0006** | 5.9505 | 8.5935 | **0.0003** |

The test compares the difference in AUC (harmonic mean population size over transfer) between the individual treatment groups.

**Supplementary Table 2: Statistical results of a general linear mixed model for yield over time for the GEN and CIP experiments.**

| | GEN experiment | | | CIP experiment | | |
|---|---|---|---|---|---|---|
| **Predictor** | $\chi^2$ | **Df** | ***P*** | $\chi^2$ | **Df** | ***P*** |
| IC | 31.34 | 3 | **<0.0001** | 16.05 | 3 | **<0.0001** |
| BN | 17.19 | 2 | **<0.0001** | 33.35 | 2 | **<0.0001** |
| Transfer | 21.46 | 1 | **<0.0001** | 48.44 | 1 | **<0.0001** |
| IC*BN | 4.47 | 6 | **0.0345** | 1.54 | 6 | 0.2151 |
| IC*Transfer | 15.40 | 3 | **<0.0001** | 5.45 | 3 | **0.0195** |
| BN*Transfer | 5.97 | 2 | **0.0145** | 2.98 | 2 | 0.0842 |
| IC*BN*Transfer | 0.73 | 6 | 0.3919 | 2.01 | 6 | 0.1565 |

The model tests the influence of drug concentration (IC), bottleneck size (BN) and time of transfer, as well as their individual interactions, on population yield. Formula: Yield ~ IC * BN * Transfer * (1 | Well on plate).

**Supplementary Table 3: Statistical results of a Tukey's HSD test for difference in yield over time for the GEN and CIP experiments.**

| | GEN experiment | | CIP experiment | |
|---|---|---|---|---|
| **Comparison** | **diff** | ***P*** | **diff** | ***P*** |
| IC20 M5-IC20 k50 | 0.19 | **<0.0001** | 0.43 | **<0.0001** |
| IC80 k50-IC20 k50 | -0.11 | **0.0058** | - 0.56 | **0.0003** |
| IC80 M5-IC20 k50 | -0.05 | 0.4816 | 0.15 | 0.0974 |
| IC80 k50-IC20 M5 | -0.31 | **<0.0001** | - 0.99 | **<0.0001** |
| IC80 M5-IC20 M5 | -0.24 | **<0.0001** | - 0.28 | **0.0001** |
| IC80 M5-IC80 k50 | 0.06 | 0.2979 | 0.71 | **<0.0001** |

The test compares the difference in mean yield between the individual treatment groups. Formula: Yield ~ Treatment.

**Supplementary Table 4: Statistical results of a Tukey's HSD test for area under curve (AUC) of growth rate from evolved populations from the GEN and CIP experiments.**

| | GEN experiment | | CIP experiment | |
|---|---|---|---|---|
| **Comparison** | **diff** | ***P*** | **diff** | ***P*** |
| IC20 M5-IC20 k50 | -0.0086 | **0.0069** | 0.0334 | **<0.0001** |
| IC80 k50-IC20 k50 | -0.0004 | 0.9976 | -0.0916 | **<0.0001** |
| IC80 M5-IC20 k50 | -0.0041 | 0.3691 | 0.0013 | 0.9956 |
| IC80 k50-IC20 M5 | 0.0082 | **0.0107** | -0.1250 | **<0.0001** |
| IC80 M5-IC20 M5 | 0.0045 | 0.3199 | -0.0321 | **<0.0001** |
| IC80 M5-IC80 k50 | -0.0037 | 0.4653 | 0.0929 | **<0.0001** |

The test compares the difference in AUC (growth rate over transfer) between the individual treatment groups. Formula: AUC ~ Treatment.

**Supplementary Table 5: Drug concentrations used to test for evolved resistance in the GEN and CIP experiments (all concentrations given in ng/ml).**

| | **IC20** | **IC80** | **MIC** | **2*MIC** | **4*MIC** | **8*MIC** | **16*MIC** |
|---|---|---|---|---|---|---|---|
| **GEN** | 380 | 500 | 600 | 1200 | 2400 | 4800 | 9600 |
| **CIP** | 15 | 40 | 60 | 120 | 240 | 480 | 960 |

**Supplementary Table 6: Linear mixed effect model results for evolved resistance for the GEN and CIP experiments.**

| | | GEN experiment | | | CIP experiment | | |
|---|---|---|---|---|---|---|---|
| **Predictor** | **Df** | **Sum Sq** | **F** | ***P*** | **Sum Sq** | **F** | ***P*** |
| IC | 3 | 0.22 | 0.71 | 0.4084 | 6.32 | 32.70 | **<0.0001** |
| BN | 2 | 0.24 | 0.76 | 0.3903 | 0.64 | 3.33 | 0.0823 |
| IC*BN | 6 | 3.53 | 11.43 | **0.0022** | 3.84 | 19.87 | **0.0002** |

The model tests the influence of drug concentration (IC), bottleneck size (BN) and their interaction on evolved resistance (AUC) to the treatment drug. Formula: AUC ~ IC * BN.

**Supplementary Table 7: Statistical results of Tukey's HSD for evolved resistance for the GEN and CIP experiments.**

| | GEN experiment | | CIP experiment | |
|---|---|---|---|---|
| **Comparison** | **diff** | ***P*** | **diff** | ***P*** |
| IC20 M5-IC20 k50 | -0.83 | **0.0292** | - 0.78 | **0.0094** |
| IC80 k50-IC20 k50 | -0.49 | 0.3110 | - 0.69 | 0.4646 |
| IC80 M5-IC20 k50 | 0.03 | 0.9994 | 0.83 | **0.0059** |
| IC80 k50-IC20 M5 | 0.34 | 0.6257 | 0.09 | 0.9975 |
| IC80 M5-IC20 M5 | 0.86 | **0.0280** | 1.61 | **<0.0001** |
| IC80 M5-IC80 k50 | 0.53 | 0.2841 | 1.52 | **0.0184** |

The test compares the difference in mean resistance (AUC) between the individual treatment groups. Formula: AUC ~ Treatment.

**Supplementary Table 8: Statistical results of a Tukey's HSD test for minimal inhibitory concentrations (MIC) of evolved populations from the GEN and CIP experiments.**

| | GEN experiment | | CIP experiment | |
|---|---|---|---|---|
| **Comparison** | **diff** | ***P*** | **diff** | ***P*** |
| IC20 M5-IC20 k50 | -6832.5 | **0.0199** | -682.5 | **0.0086** |
| IC80 k50-IC20 k50 | -4350 | 0.3089 | -540 | 0.7728 |
| IC80 M5-IC20 k50 | -2603.57 | 0.8545 | 900 | **0.0003** |
| IC80 k50-IC20 M5 | 2475 | 0.8635 | 143 | 0.9997 |
| IC80 M5-IC20 M5 | 4221.43 | 0.3834 | 1582.5 | **<0.0001** |
| IC80 M5-IC80 k50 | 1764 | 0.9760 | 1440 | **0.0091** |

The test compares the difference in MIC between the individual treatment groups. Formula: MIC ~ Treatment.

**Supplementary Table 9: Number of mutations in most frequently affected genes per treatment group at the final transfer of the GEN experiment.**

| Gene | IC20-k50 | IC20-M5 | IC80-k50 | IC80-M5 | Total |
|---|---|---|---|---|---|
| *ptsP* | 1 | 13 | 2 | 4 | 20 |
| *pmrB* | 5 | 0 | 1 | 7 | 13 |
| *parS* | 3 | 0 | 0 | 1 | 4 |
| *parR* | 1 | 0 | 3 | 1 | 5 |
| *phoQ* | 1 | 0 | 2 | 0 | 3 |
| *rbsR* | 0 | 2 | 0 | 0 | 2 |
| *HPr* | 0 | 2 | 0 | 1 | 3 |

**Supplementary Table 10: Number of mutations in most frequently affected genes per treatment group at the final transfer of CIP experiment.**

| Gene | IC20-k50 | IC20-M5 | IC80-k50 | IC80-M5 | Total |
|---|---|---|---|---|---|
| *mexS* | 4 | 0 | 0 | 10 | 14 |
| *mexZ* | 7 | 0 | 0 | 0 | 7 |
| *nfxB* | 1 | 0 | 0 | 6 | 7 |
| *copZ* | 4 | 0 | 0 | 0 | 4 |

**Supplementary Table 11: Shannon's diversity indices H and Hmax for each treatment group for the GEN and CIP experiments.**

| | GEN experiment | | CIP experiment | |
|---|---|---|---|---|
| **Treatment** | **Shannon's H** | **Hmax** | **Shannon's H** | **Hmax** |
| IC20-k50 | 3.27 | 3.46 | 2.44 | 2.81 |
| IC20-M5 | 2.04 | 2.58 | 0.97 | 1 |
| IC80-k50 | 2.85 | 3 | 1 | 1 |
| IC80-M5 | 2.05 | 2.32 | 1.58 | 1.58 |

**Supplementary Table 12: General linear model results for evolved resistance in populations carrying either *pmrB* or *ptsP* mutations.**

| **Predictor** | **Df** | **Sum Sq** | **F** | ***P*** |
|---|---|---|---|---|
| Treatment | 3 | 3.56 | 12.25 | **0.0002** |
| Gene | 1 | 3.12 | 32.24 | **<0.0001** |

The model tests the influence of treatment regimen and gene variant on evolved resistance (AUC). Formula: AUC ~ Treatment + Gene.

**Supplementary Table 13: Clones used in the three competition experiments.**

| **Competition Experiment** | **Treatment during evolution experiment** | ***pmrB* mutant from population** | ***ptsP* mutant from population** | **control** |
|---|---|---|---|---|
| 1 | IC80-k50 | D12 | A12 | PA14 |
| 2 | IC20 | A6 (k50) | B11 (M5) | PA14 |
| 3 | IC80-M5 | A5 | E7 | PA14 |

**Supplementary Table 14: Tukey HSD results for multiple pairwise-comparison of frequency of *ptsP* mutants in competition against *pmrB* mutants and treatment group.**

| **Comparison** | **diff** | **p adj.** |
|---|---|---|
| IC20 M5-IC20 k50 | - 0.19 | **0.0138** |
| IC80k50-IC20 k50 | - 0.32 | **<0.0001** |
| IC80 M5-IC20 k50 | - 0.06 | 0.8671 |
| IC80 k50-IC20 M5 | - 0.17 | **0.0352** |
| IC80 M5-IC20 M5 | - 0.14 | 0.1457 |
| IC80 M5-IC80 k50 | - 0.10 | 0.3983 |

The test compares the difference in mean frequency of *ptsP* clones in competition between the individual treatments. Formula: Frequency ~ Treatment

**Supplementary Table 15: Sequence-specific primers for amplicon sequencing (first PCR step).**

| Name | Populations | Sequence |
|---|---|---|
| pmrB-5636922-forward | A5, D12, A6 | ACACTCTTTCCCTACACGACGCTCTTCCGATCTGACCTTGCCACCGAAGACC |
| pmrB-5637204-forward | A5, G8, A6 | ACACTCTTTCCCTACACGACGCTCTTCCGATCTACCATGAACCTGCTGCTGTT |
| ptsP-394249-forward | E7 | ACACTCTTTCCCTACACGACGCTCTTCCGATCTTTCATGATCAACGACCGCTTC |
| ptsP-393899-forward | E7 | ACACTCTTTCCCTACACGACGCTCTTCCGATCTCTCGGCTCGGGCAACTC |
| ptsP-393042-forward | A12 | ACACTCTTTCCCTACACGACGCTCTTCCGATCTAACAAGCGTTCCATCGGCAA |
| ptsP-394724-forward | B11 | ACACTCTTTCCCTACACGACGCTCTTCCGATCTAGGTCGACTTCCTTTCGGTC |
| pmrB-5636922-reverse | A5, D12, A6 | GTGACTGGAGTTCAGACGTGTGCTCTTCCGATCTCACAGGGCGTAGCCGAG |
| pmrB-5637204-reverse | A5, G8, A6 | GTGACTGGAGTTCAGACGTGTGCTCTTCCGATCTGACCTCGGCCTGCACTTC |
| ptsP-394249-reverse | E7 | GTGACTGGAGTTCAGACGTGTGCTCTTCCGATCTCGGGTTGTCTTCCTTGATCGG |
| ptsP-393899-reverse | E7 | GTGACTGGAGTTCAGACGTGTGCTCTTCCGATCTGGTAGCCATCGACGATCAGG |
| ptsP-393042-reverse | A12 | GTGACTGGAGTTCAGACGTGTGCTCTTCCGATCTCCATCACCCGCCTATGGTG |
| ptsP-394724-reverse | B11 | GTGACTGGAGTTCAGACGTGTGCTCTTCCGATCTCATCGTCGACCACCTTCTTCA |

Names consist of the target gene, the position of the SNP in the genome and F or R for forward or reverse primer. The population(s) for which the primer amplifies the SNP region are listed. Overhangs of the primers are labelled in black. Sequence-specific parts of primers are in labelled in red.

**Supplementary Table 16: Sequencing primers for amplicon sequencing (second PCR step).**

| 2nd PCR | Name | Sequence |
|---|---|---|
| forward | F-1 | AATGATACGGCGACCACCGAGATCTACACAACCGCATACACTCTTTCCCTACACG |
| | F-2 | AATGATACGGCGACCACCGAGATCTACACAAGGCCTTACACTCTTTCCCTACACG |
| | F-3 | AATGATACGGCGACCACCGAGATCTACACAGAGTGTGACACTCTTTCCCTACACG |
| | F-4 | AATGATACGGCGACCACCGAGATCTACACCACAAGTCACACTCTTTCCCTACACG |
| | F-5 | AATGATACGGCGACCACCGAGATCTACACCGTTCCTAACACTCTTTCCCTACACG |
| | F-6 | AATGATACGGCGACCACCGAGATCTACACGCTTGGATACACTCTTTCCCTACACG |
| | F-7 | AATGATACGGCGACCACCGAGATCTACACGTCAACACACACTCTTTCCCTACACG |
| | F-8 | AATGATACGGCGACCACCGAGATCTACACGTCACTGAACACTCTTTCCCTACACG |
| | F-9 | AATGATACGGCGACCACCGAGATCTACACTCTCGTCAACACTCTTTCCCTACACG |
| | F-10 | AATGATACGGCGACCACCGAGATCTACACTTGGTACGACACTCTTTCCCTACACG |
| reverse | R-A | CAAGCAGAAGACGGCATACGAGATAACCGGAAGTGACTGGAGTTCAGACG |
| | R-B | CAAGCAGAAGACGGCATACGAGATAGAGTGACGTGACTGGAGTTCAGACG |
| | R-C | CAAGCAGAAGACGGCATACGAGATCAACTGGTGTGACTGGAGTTCAGACG |
| | R-D | CAAGCAGAAGACGGCATACGAGATCGTTCGTTGTGACTGGAGTTCAGACG |
| | R-E | CAAGCAGAAGACGGCATACGAGATCTGTTCACGTGACTGGAGTTCAGACG |
| | R-F | CAAGCAGAAGACGGCATACGAGATGCTTGCAAGTGACTGGAGTTCAGACG |
| | R-G | CAAGCAGAAGACGGCATACGAGATGTCAACTGGTGACTGGAGTTCAGACG |
| | R-H | CAAGCAGAAGACGGCATACGAGATTCCTCATGGTGACTGGAGTTCAGACG |
| | R-I | CAAGCAGAAGACGGCATACGAGATTCGACTAGGTGACTGGAGTTCAGACG |
| | R-J | CAAGCAGAAGACGGCATACGAGATTTGCAAGCGTGACTGGAGTTCAGACG |

Barcodes are labelled in blue. Illumina adapters are labelled in black.
