# Supplementary material for: Bottleneck size and selection level reproducibly impact evolution of antibiotic resistance
Source: Nat Ecol Evol. 2021 Jul 26;5(9):1233–42. doi: 10.1038/s41559-021-01511-2 (PMC8390372; doi:10.1038/s41559-021-01511-2)
Supplement: Supplementary file 2 — Reporting Summary [file 41559_2021_1511_MOESM2_ESM.pdf]

nature research

Corresponding author(s): Hinrich Schulenburg

Last updated by author(s): Jun 2, 2021

Reporting Summary

Nature Research wishes to improve the reproducibility of the work that we publish. This form provides structure for consistency and transparency in reporting. For further information on Nature Research policies, see our Editorial Policies and the Editorial Policy Checklist.

Statistics

For all statistical analyses, confirm that the following items are present in the figure legend, table legend, main text, or Methods section.

n/a	Confirmed	
☐	☒	The exact sample size (n) for each experimental group/condition, given as a discrete number and unit of measurement
☐	☒	A statement on whether measurements were taken from distinct samples or whether the same sample was measured repeatedly
☐	☒	The statistical test(s) used AND whether they are one- or two-sided<br>*Only common tests should be described solely by name; describe more complex techniques in the Methods section.*
☒	☐	A description of all covariates tested
☐	☒	A description of any assumptions or corrections, such as tests of normality and adjustment for multiple comparisons
☐	☒	A full description of the statistical parameters including central tendency (e.g. means) or other basic estimates (e.g. regression coefficient) AND variation (e.g. standard deviation) or associated estimates of uncertainty (e.g. confidence intervals)
☐	☒	For null hypothesis testing, the test statistic (e.g. F, t, r) with confidence intervals, effect sizes, degrees of freedom and P value noted<br>*Give P values as exact values whenever suitable.*
☒	☐	For Bayesian analysis, information on the choice of priors and Markov chain Monte Carlo settings
☐	☒	For hierarchical and complex designs, identification of the appropriate level for tests and full reporting of outcomes
☒	☐	Estimates of effect sizes (e.g. Cohen's d, Pearson's r), indicating how they were calculated

Our web collection on statistics for biologists contains articles on many of the points above.

Software and code

Policy information about availability of computer code

Data collection	Not applicable
Data analysis	R was used for general statistical analysis. Genome sequence analysis was based on the following tools: Quality and quantity of reads were checked with FastQC. Trimmomatic was used to remove sequencing adapters from the Nextera library and to filter out low quality reads. High quality reads were mapped to the UCBPP_PA14 reference genome with the software bwa. The generated .bam files were scanned for SNPs, insertions and deletions using the variant calling programs FreeBayes, PinDel and VarScan. The resulting output files were filtered for duplicates, ancestral variants, and variants found in the evolved controls using R and additionally checked by visually inspecting the called genome positions provided by the .bam file in the IGV genome browser. The detected variants were annotated with the help of SnpEff and the Pseudomonas database (available at http://pseudomonas.com). The R package "ggmuller" was used to generate Muller plots.

For manuscripts utilizing custom algorithms or software that are central to the research but not yet described in published literature, software must be made available to editors and reviewers. We strongly encourage code deposition in a community repository (e.g. GitHub). See the Nature Research guidelines for submitting code & software for further information.

Data

Policy information about availability of data

All manuscripts must include a data availability statement. This statement should provide the following information, where applicable:

- Accession codes, unique identifiers, or web links for publicly available datasets
- A list of figures that have associated raw data
- A description of any restrictions on data availability

Whole genome sequencing data for bacterial populations are available from Genbank at the National Center for Biotechnology Information (NCBI, USA) under

accession numbers PRJNA725112 and PRJNA725351. The experimental data are available from Dryad (doi:10.5061/dryad.dncjsxm06) and also the supplementary Source Data Tables.

Field-specific reporting

Please select the one below that is the best fit for your research. If you are not sure, read the appropriate sections before making your selection.

☐ Life sciences ☐ Behavioural & social sciences ☒ Ecological, evolutionary & environmental sciences

For a reference copy of the document with all sections, see nature.com/documents/nr-reporting-summary-flat.pdf

Ecological, evolutionary & environmental sciences study design

All studies must disclose on these points even when the disclosure is negative.

Study description	Experimental evolution, whole genome sequence analysis and competition experiments were used to assess the influence of bottleneck size and antibiotic induced selection on drug resistance evolution in Pseudomonas aeruginosa.
Research sample	Experimentally evolved bacteria of the strain PA14 of Pseudomonas aeruginosa
Sampling strategy	2 independent evolution experiments were performed, based on identical fully factorial design, including 2 factors, one with 3 levels (antibiotic concentration) and the other with two levels (bottleneck size), plus additionally 8 replicate populations per treatment combination. The evolution experiments were performed by serial dilution over 16 transfer periods.
Data collection	Evolving bacterial populations were sampled every other transfer period
Timing and spatial scale	Evolving bacterial populations were sampled every other transfer period to characterize the temporal dynamics of evolutionary change
Data exclusions	All data points were included in our analysis. For the temporal genomic analysis, we excluded treatments without any genomic changes (1 treatment in 1 of the 2 experiments) and treatments, for which all but one replicate population went extinct (1 treatment in 1 of the experiments)
Reproducibility	2 fully independent evolution experiments were performed, each with 8 replicates per treatment combination
Randomization	Treatment combinations were randomly distributed across 96-well plates
Blinding	Treatments were masked to minimize observer bias

Did the study involve field work? ☐ Yes ☒ No

Reporting for specific materials, systems and methods

We require information from authors about some types of materials, experimental systems and methods used in many studies. Here, indicate whether each material, system or method listed is relevant to your study. If you are not sure if a list item applies to your research, read the appropriate section before selecting a response.

Materials & experimental systems

n/a	Involved in the study
☒	☐ Antibodies
☒	☐ Eukaryotic cell lines
☒	☐ Palaeontology and archaeology
☒	☐ Animals and other organisms
☒	☐ Human research participants
☒	☐ Clinical data
☒	☐ Dual use research of concern

Methods

n/a	Involved in the study
☒	☐ ChIP-seq
☒	☐ Flow cytometry
☒	☐ MRI-based neuroimaging
