# Supplementary material for: Bottleneck size and selection level reproducibly impact evolution of antibiotic resistance
Source: Nat Ecol Evol. 2021 Jul 26;5(9):1233–42. doi: 10.1038/s41559-021-01511-2 (PMC8390372; doi:10.1038/s41559-021-01511-2)
Supplement: Supplementary file 3 — Peer Review Information [file 41559_2021_1511_MOESM3_ESM.pdf]

natureresearch

Peer Review Information

Journal: Nature Ecology & Evolution
Manuscript Title: Bottleneck size and selection level reproducibly impact antibiotic resistance evolution
Corresponding author name(s): Hinrich Schulenburg

Editorial Notes:

Reviewer Comments & Decisions:

Decision Letter, initial version:

16th February 2021

Dear Professor Schulenburg,

Your Article entitled "Bottleneck size and selection level reproducibly impact antibiotic resistance evolution" has now been seen by 3 reviewers, whose comments are attached. In the light of their advice, we have decided that we cannot offer to publish your manuscript in Nature Ecology & Evolution.

From the reports, you will see that while they find your work of some potential interest, the reviewers raise concerns about the advance your findings represent over earlier work and the strength of the novel conclusions that can be drawn at this stage. We feel that these criticisms are sufficiently important as to preclude publication of your work in Nature Ecology & Evolution.

I am sorry that we cannot be more positive on this occasion, but hope that you find the reviewers' comments helpful when preparing your paper for resubmission elsewhere. If you would like to consider transferring the manuscript along with the reviewers' reports to one of our sister journals, you can do so using the link at the bottom of this email.

[REDACTED]

Reviewers Comments:

Reviewer #1 (Remarks to the Author):

The authors present novel results on a fundamental problem, i.e. how bacterial population bottleneck size and antibiotic concentration affect the evolution of resistance. They performed a short (100 generation) evolution experiment with Pseudomonas aeruginosa, challenged with 2 antibiotics (gentamicin and ciprofloxacin), administered at 3 levels each, and applying 2 bottleneck sizes. Based on fitness assays and genome analyses of the evolved populations, they find that both drug concentration and bottleneck size affect resistance pathways. Specifically, the authors conclude that smaller bottlenecks cause greater divergence and resistance evolution is favored both under high antibiotic levels with large bottlenecks and low antibiotic levels with small bottlenecks. The impact of population bottlenecks on evolutionary trajectories is an important problem, given the natural role of bottlenecks in pathogen transmission and selection and the fact that we have limited understanding of this fundamental factor. However, I feel that the advance made by the study of Mahrt et al. is modest, among others due to lack of information of key variables and problems with the interpretation and presentation of the data.

Major comments

First, the authors present and interpret yield as measure of fitness in the absence of the drug, whereas it is unclear how this measure can be interpreted in terms of fitness in the unstructured populations under their culture regime. Growth rate would be a more relevant fitness measure, and it is unclear whether and how yield correlates with growth rate under these conditions – leaving the possibility that the observed high yield under the IC20-M5 treatment actually reflects low fitness. Similarly, the dose-response curves used to measure resistance were also based on OD point measurements rather than growth rates, hence are confounded by yield and possibly less by MIC (minimal inhibitory concentration) value. Second, it is dissatisfying that no genomic changes were detected in any of the 8 Cip IC20-M5 populations, while this treatment shows the largest response in yield and resistance. The authors simply conclude that "this suggests that phenotypic responses are sufficient to counter the low selective constraints imposed", without discussing possible mechanisms or providing minimal information about the heritability of this response. A possible problem here seems that the authors sequenced metagenomes, not clones, which makes it hard to detect chromosomal rearrangements in minority genotypes, which have been found to play a role in antibiotic resistance (e.g. causing heteroresistance). Third, where the different bottleneck treatments result in different mutational targets, as for GEN, it would be informative to discuss the role of mutation bias: do mutations in small-bottleneck targets (e.g. pmrB) perhaps occur at higher rate (e.g. nonsense mutations, indels in repeat regions or transitions instead of transversions) than mutations found for large bottlenecks (e.g. ptsP)?

Other comments

1. Fig. 1a and c show relative yield, while in the text cumulative yield is mentioned. Is this the same? It is also unclear what the boxes in the panels mean.
2. Line 106-107: "weak bottlenecks consistently favoured variants in only few genes", for which Fig. 2 shows little support, with 5 versus 7 genes affected under Gen selection.
3. Line 131-132: "In consistency with the GEN experiment, weak bottlenecks led to variants in much fewer genes (Fig. 2b)", again for which Fig. 2b provides little support, because there are no data for comparing bottleneck size, not for IC20 (IC20-M5 data lacking) nor for IC80 where only 1 population is shown for k50 bottleneck.
4. Fig. 4b: The figure says "Competitive fitness", but instead shows competitor frequency at the end of

competition. Why not present easy-to-interpret relative fitness estimates?

Reviewer #2 (Remarks to the Author):

The manuscript explores the role of population bottlenecks and selection intensity in the evolution of antibiotic resistance. The authors employ a novel flow-cytometry method to manipulate bottleneck intensity, and then experimentally evolve resistance to two doses of two antibiotics. The author characterize adaptation at both the phenotypic and genomic scales. The authors find that the evolution of resistance can be favoured by either strong selection and weak bottlenecks, or weak selection and strong bottlenecks. The authors discuss their results in relation to evolutionary theory, published manuscripts, and clinical scenarios.

The manuscript is well written, the methods are clear, and the data visualisations are excellent throughout. However I have reservations about how the authors relate their experiment design and findings to both theory and other experiments.

The problem with this design is that it has decoupled population size and bottleneck intensity. As such both the predictions and the data interpretations are not as straightforward as the authors suggest. The theory the authors cite in the introduction is about population size; big populations have a greater supply of beneficial mutations, therefore faster, more parallel adaptation. If bottleneck size were the only treatment, this theory could be applied directly, as it would correlate with population size. But as selection intensity also affects population size, the predictive power and relevance of bottleneck size is far less clear. For example, the weak-selection, strong bottleneck treatment (IC20 K50) creates a decent population size and it is not “unexpected” that resistance can evolve quite readily under these conditions. Likewise, the lack of adaptation in the populations with a greater population size experiencing the same weak selection is also not unexpected. The ancestor will be harder to out-compete, as well as the greater mutation supply will open other routes of adaptation, not just evolving resistance.

I am not suggesting that the manuscript is fundamentally flawed, but I would suggest the manuscript would benefit from a more nuanced setup and interpretation. Although the authors do discuss the role of population size at length in the discussion, I feel it needs greater prominence throughout the manuscript.

Minor points

- Perhaps the authors could calculate the harmonic mean population size, which would incorporate both population size and bottleneck size. This may make the manuscript tie-in better with what theory would predict and which of their findings are unexpected.
- The language of strong vs wide bottlenecks is odd. These are not contradictory terms, and either strong/weak or narrow/wide would be more consistent.
- Line 12-14: what is lacking from the literature is the interaction between bottlenecks and selection intensity, and I think this sentence should be rephrased to reflect the manuscripts novelty. In general I think the abstract and introduction could do a better job emphasising this aspect of the work.
- Line 31-33: I think this is a debatable point, so I would change this to “may contribute” or “could be contributing”. The citations the authors provide to support this statement discuss the role of

environmental contamination but provide no proof of its importance.
- Line 37. “In theory.” I think there are enough published, empirical studies to cut that phrase.
- Line 39-41 . Again the interaction is what is missing, as the author cite more than ten papers exploring the role of bottlenecks in adaptation.

Reviewer #3 (Remarks to the Author):

Interesting and well performed study addressing the impact of bottlenecks imposed by population size and selection levels on the evolutionary paths to antimicrobial resistance to two antibiotic classes in P. aeruginosa model. Some comments for the authors consideration:

1. Population size is assessed by flow cytometry. How does it reflect cell viability?

2. Discussion on the observed resistance mechanisms should be compared with existing data from other studies (for example Cabot et al AAC 2016 for fluoroquinolones or lópez-causapé AAC 2018 for aminoglycosides).

3. Might the high population size still be too small for infrequent highly benefitial mutations (such as highly specific gain of function mutations)?

4. Related to this, it is surprising that target mutations (such as gyrA for cip and fusA1 for gen) are not selected under high level selective pressure.

5. Discussion, lines 242-245. It is an interesting issue, I would have expected perhaps the opposite, since phenotypic/inducible/adaptive resistance to aminoglycosides is well established in contrast to fluoroquinolone resistance

****

**Although we cannot offer to publish your paper in Nature Ecology & Evolution, the work may be appropriate for another journal in the Nature Research portfolio. If you wish to explore suitable journals and transfer your manuscript to a journal of your choice, please use our manuscript transfer portal. If you transfer to Nature-branded journals or to the Communications journals, you will not have to re-supply manuscript metadata and files. This link can only be used once and remains active until used.
All Nature Research journals are editorially independent, and the decision to consider your manuscript will be taken by their own editorial staff. For more information, please see our manuscript transfer FAQ page. Note that any decision to opt in to In Review at the original journal is not sent to the receiving journal on transfer.

You can opt in to <i>In Review</i> at receiving journals that support this service by choosing to modify your manuscript on transfer. In Review is available for primary research manuscript types only.

** For Nature Research general information and news for authors, see http://npg.nature.com/authors.

Author Rebuttal to Initial comments

Reponse to reviewers' comments

Reviewers' comments in grey and italics; our response is given in blue

Reviewers Comments:

Reviewer #1 (Remarks to the Author):

The authors present novel results on a fundamental problem, i.e. how bacterial population bottleneck size and antibiotic concentration affect the evolution of resistance. They performed a short (100 generation) evolution experiment with Pseudomonas aeruginosa, challenged with 2 antibiotics (gentamicin and ciprofloxacin), administered at 3 levels each, and applying 2 bottleneck sizes. Based on fitness assays and genome analyses of the evolved populations, they find that both drug concentration and bottleneck size affect resistance pathways. Specifically, the authors conclude that smaller bottlenecks cause greater divergence and resistance evolution is favored both under high antibiotic levels with large bottlenecks and low antibiotic levels with small bottlenecks. The impact of population bottlenecks on evolutionary trajectories is an important problem, given the natural role of bottlenecks in pathogen transmission and selection and the fact that we have limited understanding of this fundamental factor. However, I feel that the advance made by the study of Mahrt et al. is modest, among others due to lack of information of key variables and problems with the interpretation and presentation of the data.

Our response: Many thanks for the detailed evaluation of our manuscript and for emphasizing the general importance of our manuscript's focus. We politely disagree that the advance is modest. Our work remains one of the very few studies, in which the consequences of population bottlenecks is revealed on antibiotic resistance evolution. This is a highly relevant topic, as bottlenecks occur repeatedly during infection. By using an experimental approach and obtaining highly consistent results across two fully independent evolution experiments, our findings of the consequences of bottlenecks on resistance evolution are robust and meaningful.

Major comments

First, the authors present and interpret yield as measure of fitness in the absence of the drug, whereas it is unclear how this measure can be interpreted in terms of fitness in the unstructured populations under their culture regime. Growth rate would be a more relevant fitness measure, and it is unclear whether and how yield correlates with growth rate under these conditions – leaving the possibility that the observed high yield under the IC20-M5 treatment actually reflects low fitness.

Our response: Many thanks for this assessment. We measured growth characteristics for all treatments continuously during the evolution experiments. Please note that evolution experiments were run in plate-readers, in which we measured OD in 15-min intervals throughout all seasons. Therefore, we possess the data to look at other growth characteristics, such as growth rate. However, we politely disagree that the rate of growth is necessarily the most important and relevant parameter defining bacterial fitness. In particular, when there is little growth – as observed in many of the antibiotic treatments, especially at the beginning of the experiment – then the estimation of growth rate is highly unreliable. For this reason, we chose to focus on final yield, which was always determined with the help of a cytometer as absolute counts of viable cells (and not based on OD), thus yielding reliable and – in

our opinion – highly informative data. Nevertheless, we would have the data to calculate growth rate and could add such information to a revised manuscript.

Similarly, the dose-response curves used to measure resistance were also based on OD point measurements rather than growth rates, hence are confounded by yield and possibly less by MIC (minimal inhibitory concentration) value.

Our response: For inference of dose response curves, bacterial populations were exposed to different concentrations of an antibiotic and then grown in a plate reader with OD measurements taken in 15-min intervals. As above, we would have the data to calculate growth rates. However, similar to our argument above, the calculation of growth rate is less reliable when there is little growth, as under the high antibiotic concentrations. Therefore, we consider the area-under-the-curve (AUC) and thus bacterial numbers across the entire growth season to represent a more reliable measure of the bacteria's response to antibiotic. For us, it was important to use a measure that can be reliably inferred across the different populations from the distinct treatment groups and that therefore is a measure that ensures comparability. We strongly believe that AUC fulfils this criterion of comparability across treatment groups and therefore allows us to assess whether changes in resistance occurred. Nevertheless, we do have the data to calculate growth rates and could add such results to a revised manuscript.

We then agree with the reviewer that OD measurements could potentially not reflect cell numbers. In this case, we still decided to use OD measurements for some of the measurements as a proxy, in order to ensure feasibility of our study (which included large sample numbers) and also because our previous more detailed assessment of the correlation between cfu counts and OD measurements suggested that OD measures do provide a reliable proxy for cell numbers. See for example our previous publications: Barbosa et al. 2017 Mol Biol Evol and Barbosa et al. 2018 PLoS Biology.

Second, it is dissatisfying that no genomic changes were detected in any of the 8 Cip IC20-M5 populations, while this treatment shows the largest response in yield and resistance. The authors simply conclude that "this suggests that phenotypic responses are sufficient to counter the low selective constraints imposed", without discussing possible mechanisms or providing minimal information about the heritability of this response. A possible problem here seems that the authors sequenced metagenomes, not clones, which makes it hard to detect chromosomal rearrangements in minority genotypes, which have been found to play a role in antibiotic resistance (e.g. causing heteroresistance).

Our response: We agree with the reviewer that it would have been nice to identify a genetic basis for the phenotypic change observed for the CIP IC20-M5 populations. Unfortunately, this was not the case. We politely disagree that this is dissatisfying. This is the result. Please note that the populations from this treatment only showed a large phenotypic change in yield, but actually no change in resistance.

We further politely disagree that it is a problem that we have sequenced the genomes of entire populations rather than individual clones. Population genome sequencing allows us to assess changes in allele frequencies at higher resolution than sequencing 1-3 clones, as is usually done in similar studies. The focus on only 1-3 clones would not permit to identify mutations in low frequency genotypes. Our approach does allow this, especially as we usually have an average coverage of >100x for the sequenced

populations. We do agree that certain types of structural variations cannot be identified using this approach. However, this does not apply to all structural variation, as we and others have demonstrated in the past (e.g., relevant CNVs can be reliably detected; see our previous work in Papkou et al. 2019 PNAS or Lähnemann et al. 2014 Genome Biol Evol). Overall, the population genomics approach has more advantages than disadvantages over sequencing of only 1-3 clones, especially for the characterization of relevant allele frequency alterations. We see this as a strength of our genomic analysis.

Most importantly, for the overall interpretation of our results across treatments, it is not relevant that we may have missed certain types of structural variation. There is no doubt that the molecular basis of the phenotypic changes in this particular IC20-M5 treatment is distinct from the other treatments, for which exactly the same population genomics approach revealed changes in a variety of different genes.

Third, where the different bottleneck treatments result in different mutational targets, as for GEN, it would be informative to discuss the role of mutation bias: do mutations in small-bottleneck targets (e.g. pmrB) perhaps occur at higher rate (e.g. nonsense mutations, indels in repeat regions or transitions instead of transversions) than mutations found for large bottlenecks (e.g. ptsP)?

Our response: We thank the reviewer for this valuable comment. This is a point, which we would be happy to address and discuss in a revised manuscript. Our data would allow us to assess this point. It would then be an interesting add-on to our current results. However, it will not change any of the main conclusions on the dynamics of evolutionary changes presented in the current manuscript.

Other comments

1. Fig. 1a and c show relative yield, while in the text cumulative yield is mentioned. Is this the same? It is also unclear what the boxes in the panels mean.

Our response: Many thanks for this comment. It is the same and we can use more precise wording in our revised manuscript. We can also easily explain the boxes in the panels.

2. Line 106-107: "weak bottlenecks consistently favoured variants in only few genes", for which Fig. 2 shows little support, with 5 versus 7 genes affected under Gen selection.
3. Line 131-132: "In consistency with the GEN experiment, weak bottlenecks led to variants in much fewer genes (Fig. 2b)", again for which Fig. 2b provides little support, because there are no data for comparing bottleneck size, not for IC20 (IC20-M5 data lacking) nor for IC80 where only 1 population is shown for k50 bottleneck.

Our response: Many thanks for these related comments. We agree that we should have been more careful in our wording.
4. Fig. 4b: The figure says "Competitive fitness", but instead shows competitor frequency at the end of competition. Why not present easy-to-interpret relative fitness estimates?
Our response: In a revised manuscript, we can easily present relative fitness estimates.

Reviewer #2 (Remarks to the Author):

The manuscript explores the role of population bottlenecks and selection intensity in the evolution of antibiotic resistance. The authors employ a novel flow-cytometry method to manipulate bottleneck intensity, and then experimentally evolve resistance to two doses of two antibiotics. The author characterize adaptation at both the phenotypic and genomic scales. The authors find that the evolution of resistance can be favoured by either strong selection and weak bottlenecks, or weak selection and strong bottlenecks. The authors discuss their results in relation to evolutionary theory, published manuscripts, and clinical scenarios.

The manuscript is well written, the methods are clear, and the data visualisations are excellent throughout. However I have reservations about how the authors relate their experiment design and findings to both theory and other experiments.

The problem with this design is that it has decoupled population size and bottleneck intensity. As such both the predictions and the data interpretations are not as straightforward as the authors suggest. The theory the authors cite in the introduction is about population size; big populations have a greater supply of beneficial mutations, therefore faster, more parallel adaptation. If bottleneck size were the only treatment, this theory could be applied directly, as it would correlate with population size. But as selection intensity also affects population size, the predictive power and relevance of bottleneck size is far less clear. For example, the weak-selection, strong bottleneck treatment (IC20 K50) creates a decent population size and it is not "unexpected" that resistance can evolve quite readily under these conditions. Likewise, the lack of adaptation in the populations with a greater population size experiencing the same weak selection is also not unexpected. The ancestor will be harder to out-compete, as well as the greater mutation supply will open other routes of adaptation, not just evolving resistance.

I am not suggesting that the manuscript is fundamentally flawed, but I would suggest the manuscript would benefit from a more nuanced setup and interpretation. Although the authors do discuss the role of population size at length in the discussion, I feel it needs greater prominence throughout the manuscript.

Our response: This is a very important point and we agree with all concerns. In fact, it is something, which we intensively discussed while setting up the project and designing the experiment. We now realized that we should have explained our rationale in more detail. Our aim was to assess to what extent bottlenecks – which are common during infection – affect the pathogen's ability to respond to selection. As the reviewer correctly points out, antibiotics themselves affect population size. The same actually applies to any other selective constraint. Therefore, the evolutionary/adaptive consequences of a bottleneck cannot be viewed alone in isolation, but it always needs to be related to the relevant selective constraint that challenges the evolving populations after having or not having experienced the bottleneck. In the end, this is the reason why we included two distinct antibiotic concentrations in our

experimental design, because this then allows us to assess whether the bottleneck alone or its interaction with the selective constraint determines the ability of the bacteria to adapt.

We believe that this design is not a problem, but actually a particular strength of our study. By varying both, we can specifically assess which aspects are affected by a strong bottleneck alone. This would not have been possible if we had used a single selective constraint (i.e., only a single antibiotic concentration). Please note that a particular value of our work is that we could consistently identify the evolutionary consequences of a strong bottleneck across two fully independent experiments, highlighting that the results are robust and meaningful.

Minor points

- Perhaps the authors could calculate the harmonic mean population size, which would incorporate both population size and bottleneck size. This may make the manuscript tie-in better with what theory would predict and which of their findings are unexpected.

Our response: This is an excellent suggestion and this is something we should have done. We are happy to do so in the revised manuscript.

- The language of strong vs wide bottlenecks is odd. These are not contradictory terms, and either strong/weak or narrow/wide would be more consistent.
- Line 12-14: what is lacking from the literature is the interaction between bottlenecks and selection intensity, and I think this sentence should be rephrased to reflect the manuscripts novelty. In general I think the abstract and introduction could do a better job emphasising this aspect of the work.
- Line 31-33: I think this is a debatable point, so I would change this to "may contribute" or "could be contributing". The citations the authors provide to support this statement discuss the role of environmental contamination but provide no proof of its importance.
- Line 37. "In theory." I think there are enough published, empirical studies to cut that phrase.
- Line 39-41 . Again the interaction is what is missing, as the author cite more than ten papers exploring the role of bottlenecks in adaptation.
Our response: Many thanks for these comments. We would be happy to follow the advice, in order to improve presentation and discussion of our data.

Reviewer #3 (Remarks to the Author):

Interesting and well performed study addressing the impact of bottlenecks imposed by population size and selection levels on the evolutionary paths to antimicrobial resistance to two antibiotic classes in P. aeruginosa model. Some comments for the authors consideration:

1. Population size is assessed by flow cytometry. How does it reflect cell viability?
Our response: Many thanks for this comment. We always used a live-dead stain on the bacteria prepared for flow cytometry. Thus, the cytometry results, which we give in our manuscript, always refer

to the viable cells. We realized that this was not well described, which are happy to improve in a revised version of the manuscript.

2. Discussion on the observed resistance mechanisms should be compared with existing data from other studies (for example Cabot et al AAC 2016 for fluoroquinolones or lópez-causapé AAC 2018 for aminoglycosides).

Our response: This is an interesting point. Because of space limitations, we focused our discussion on the molecular mechanisms of resistance in consideration of the current literature to the two genes *pmrB* and *ptsP*. These are the two genes, which we also studied in more detail. Nevertheless, this discussion could be easily extended to other genes and mechanisms, as suggested by the reviewer.

3. Might the high population size still be too small for infrequent highly benefitial mutations (such as highly specific gain of function mutations)?
4. Related to this, it is surprising that target mutations (such as gyrA for cip and fusA1 for gen) are not selected under high level selective pressure.

Our response: Many thanks for these two related comments. In order to ensure feasibility, we chose antibiotic concentrations below the MIC – otherwise, the study of adaptive responses is difficult and often impossible. As noted by others previously, such lower antibiotic concentrations often favor mutations in other genes than the target genes. This is indeed an interesting point, which however does not affect the overall findings and interpretation of the results (i.e., a difference in evolutionary adaptation in populations with a strong or mild bottleneck), even if it would be worth a comment in the discussion. Please also note that high population size was sufficient for favoring emergence of high resistance mutations, as documented by the large increase in resistance for the IC80-M5 treatments, consistently observed across the two independent evolution experiments.

5. Discussion, lines 242-245. It is an interesting issue, I would have expected perhaps the opposite, since phenotypic/inducible/adaptive resistance to aminoglycosides is well established in contrast to fluoroquinolone resistance

Our response: We agree that this result may not have been expected *a priori*. This is the reason, why we specifically addressed this result in the discussion in lines 242-245 and present possible explanation for the findings made.

Decision Letter, first revision:

11th March 2021

Dear Hinrich,

Thank you for your letter asking us to reconsider our decision on your Article entitled "Bottleneck size and selection level reproducibly impact antibiotic resistance evolution". After careful consideration we have decided that we would be willing to consider a revised version of your manuscript.

Along with your revised manuscript, you should also submit a separate point-by-point response to all of the concerns raised by the reviewers, in each case describing what changes have been made to the manuscript or, alternatively, if no action has been taken, providing a compelling argument for why that is the case. If we feel that a substantial attempt has been made to address the reviewers' comments, this response will be sent back to the reviewers - along with the revised manuscript - so that they can judge whether their concerns have been addressed satisfactorily or otherwise.

I should stress, however, that we would be reluctant to trouble our reviewers again unless we thought that their comments had been addressed in full.

When revising your paper:

- ensure it complies with our format requirements for Articles as set out in our guide to authors at www.nature.com/natecolevol/authors/index.html

- state in a cover note the length of the text, methods and legends; the number of references and the number of display items.

Please ensure that all correspondence is marked with your Nature Ecology & Evolution reference number in the subject line.

Please use the following link to submit your revised manuscript:

[REDACTED]

I would appreciate it if you could tell me if you think you will be able to submit a revised manuscript, and also the likely timescale.

I look forward to hearing from you soon.

[REDACTED]

Author Rebuttal, first revision:

Reponse to reviewers' comments

Reviewers' comments in grey and italics; our response is given in blue. We additionally provide a manuscript file, in which all changes are highlighted in red. All below line numbers refer to this additional manuscript file.

Reviewers Comments:

Reviewer #1 (Remarks to the Author):

The authors present novel results on a fundamental problem, i.e. how bacterial population bottleneck size and antibiotic concentration affect the evolution of resistance. They performed a short (100 generation) evolution experiment with Pseudomonas aeruginosa, challenged with 2 antibiotics (gentamicin and ciprofloxacin), administered at 3 levels each, and applying 2 bottleneck sizes. Based on fitness assays and genome analyses of the evolved populations, they find that both drug concentration and bottleneck size affect resistance pathways. Specifically, the authors conclude that smaller bottlenecks cause greater divergence and resistance evolution is favored both under high antibiotic levels with large bottlenecks and low antibiotic levels with small bottlenecks. The impact of population bottlenecks on evolutionary trajectories is an important problem, given the natural role of bottlenecks in pathogen transmission and selection and the fact that we have limited understanding of this fundamental factor. However, I feel that the advance made by the study of Mahrt et al. is modest, among others due to lack of information of key variables and problems with the interpretation and presentation of the data.

Our response: Many thanks for the detailed evaluation of our manuscript and for emphasizing the general importance of our manuscript's focus. We politely disagree that the advance is modest. Our work remains one of the very few studies, in which the consequences of population bottlenecks is revealed on antibiotic resistance evolution. This is a highly relevant topic, as bottlenecks occur repeatedly during infection. By using an experimental approach and obtaining highly consistent results across two fully independent evolution experiments, our findings of the consequences of bottlenecks on resistance evolution are robust and meaningful.

Major comments

First, the authors present and interpret yield as measure of fitness in the absence of the drug, whereas it is unclear how this measure can be interpreted in terms of fitness in the unstructured populations under their culture regime. Growth rate would be a more relevant fitness measure, and it is unclear whether and how yield correlates with growth rate under these conditions – leaving the possibility that the observed high yield under the IC20-M5 treatment actually reflects low fitness.

Our response: Many thanks for this assessment. We measured growth characteristics for all treatments continuously during the evolution experiments. Please note that evolution experiments were run in

plate-readers, in which we measured OD in 15-min intervals throughout all seasons. Therefore, we possess the data to look at specific growth characteristics, such as growth rate. However, we politely disagree that the rate of growth is necessarily the most important and relevant parameter defining bacterial fitness. In particular, when there is little growth – as observed in many of the antibiotic treatments, especially at the beginning of the experiment – then the estimation of growth rate is highly unreliable. For this reason, we chose to focus on final yield, which was always determined with the help of a cytometer as absolute counts of viable cells (and not based on OD), thus yielding reliable and – in our opinion – highly informative data. However, we now noted a contradiction in the description of how yield was determined, which was correctly explained in the methods section but not in the results section of the previous manuscript. We apologize for this mistake, which has now been corrected throughout the manuscript, emphasizing that yield was always calculated from the counts of viable cells, inferred from flow cytometry. Moreover, as we have the data, we now additionally followed the reviewer's advice and calculated growth rates for the evolving populations.

In detail, we used our OD measurements and the software GrowthRates (v. 4.3, https://sourceforge.net/projects/growthrates/files/) to calculate the growth rates of every growth period. We then determined the AUC of growth rate across time in R, thus providing a similar integrative measure of bacterial fitness across the evolution experiment, as previously obtained for final yield. The new results are presented in Extended Data Figure 4. For the CIP experiment, populations of IC20-M5 had the overall highest growth rate, which is in accordance with the results for yield. For the GEN experiment, the results for growth rate and yield vary. For example, the IC20-M5 treatment had the highest yield but the lowest growth rate, indicating that the populations achieved high cell counts not by the replication rate alone, but also other factors that enhance the bacteria's competitiveness and as further evaluated with the competition experiments performed. In the revised manuscript, we now explain the new approach and corresponding data in the Results (lines 76-80 and 85-92) and evaluate the implications in the discussion (see lines 300-305). We also explain the methods for calculating growth rates in the Methods section (lines 575-580). Furthermore, we explain in the Methods section that final yield was inferred with a cytometer as absolute counts of viable cells and therefore considered as a reliable and informative proxy for bacterial fitness (see lines 525-530 and 567-575). To enhance clarity, we present these methods under a new header "Assessment of bacterial fitness" (line 567). To avoid misunderstandings, we also rephrased the description of the yield results in the legend to Figure 1, where we now emphasize that final yield was determined by the absolute number of viable cells (lines 110-113). The results of the statistical analyses are provided in Extended Data Tables C and D.

Similarly, the dose-response curves used to measure resistance were also based on OD point measurements rather than growth rates, hence are confounded by yield and possibly less by MIC (minimal inhibitory concentration) value.

Our response: Our assessment of resistance changes generally follows standard procedure of medical diagnostics. This standard procedure, as for example in the widely used Vitek2 approach (bioMérieux Ltd), exposes a bacterial strain in broth culture to different antibiotic concentration, to identify the minimum concentration without growth (MIC), measured as turbidity of the culture and thus OD. A similar approach on Agar plates (e.g., Etests) also relies on the absence of bacterial colonies to identify the MIC. Therefore, we do consider our measure of resistance changes to be reliable and informative.

Nevertheless, we realized that we could additionally show directly the minimum concentration without growth – in consistency with standard diagnostic procedure. Our current measure is based on the AUC across the entire dose response curve. It represents an integrative measure of resistance, which captures the bacterial response across the included range of concentrations and which we therefore consider to be highly sensitive and informative measure of resistance. Still, we can use the dose response curve to identify the specific concentration of no growth, yielding a more conservative measure of resistance. The results for this measure are highly consistent with the AUC results. They are now included in Extended Data Figure 6 of the revised manuscript. The methods are described in the Methods section (lines 588-595), the results described in the Results section (lines 93-98), and the statistical results provided in Extended Data table H.

Second, it is dissatisfying that no genomic changes were detected in any of the 8 Cip IC20-M5 populations, while this treatment shows the largest response in yield and resistance. The authors simply conclude that "this suggests that phenotypic responses are sufficient to counter the low selective constraints imposed", without discussing possible mechanisms or providing minimal information about the heritability of this response. A possible problem here seems that the authors sequenced metagenomes, not clones, which makes it hard to detect chromosomal rearrangements in minority genotypes, which have been found to play a role in antibiotic resistance (e.g. causing heteroresistance).

Our response: We agree with the reviewer that it would have been nice to identify a genetic basis for the phenotypic change observed for the CIP IC20-M5 populations. Unfortunately, this was not the case. We politely disagree that this is dissatisfying. This is the result. Please note that the populations from this treatment only showed a large phenotypic change in yield, but actually no change in resistance.

We further politely disagree that it is a problem that we have sequenced the genomes of entire populations rather than individual clones. Population genome sequencing allows us to assess changes in allele frequencies at higher resolution than sequencing 1-3 clones, as is usually done in similar studies. The focus on only 1-3 clones would not permit to identify mutations in low frequency genotypes. Our approach does allow this, especially as we usually have an average coverage of >100x for the sequenced populations. We do agree that certain types of structural variations cannot be identified using this approach. However, this does not apply to all structural variation, as we and others have demonstrated in the past, including reliable detection of relevant CNVs (see our previous work in Papkou et al. 2019 PNAS or Lähnemann et al. 2014 Genome Biol Evol). Overall, the population genomics approach has more advantages than disadvantages over sequencing of only 1-3 clones, especially for the characterization of relevant allele frequency changes. We see this as a strength of our genomic analysis.

Most importantly, for the overall interpretation of our results across treatments, it is not relevant that we may have missed certain types of structural variation. There is no doubt that the molecular basis of the phenotypic changes in this particular IC20-M5 treatment is distinct from the other treatments, for which exactly the same population genomics approach revealed changes in a variety of different genes.

In the revised manuscript, we now describe our reasoning for using whole population genome sequencing (lines 596-598). In the Results section, we further emphasize that the results for the IC20-M5 treatment is clearly distinct to the other treatments, thus indicating a different type of response in comparison to the other treatments (lines 164-167).

Third, where the different bottleneck treatments result in different mutational targets, as for GEN, it would be informative to discuss the role of mutation bias: do mutations in small-bottleneck targets (e.g. pmrB) perhaps occur at higher rate (e.g. nonsense mutations, indels in repeat regions or transitions instead of transversions) than mutations found for large bottlenecks (e.g. ptsP)?

Our response: We thank the reviewer for this valuable comment. We now assessed the total number of variants in the most commonly mutated genes of the final transfer of both data sets. The results are summarized in the Extended Data Tables I and J. Please note that this type of data is not really suited for the assessment of mutational biases, because the presence of a particular variant in this data is a consequence of both mutation and spread of the mutation within the population to detectable frequencies. Nevertheless, this assessment highlights that for the GEN treatment, it is rather *ptsP* variants which are found more often across the treatments than variants in any of the other genes, including *pmrB*. Thus, this result additionally supports our conclusion that the differential prevalence of *ptsP* versus *pmrB* variants across treatment groups is best explained by their difference in competitiveness under different antibiotic concentrations, which in turn results from both the resistance level provided and the associated growth cost. These additional insights are now explained in the Results section (lines 135-138 and 196 and following).

Other comments

1. Fig. 1a and c show relative yield, while in the text cumulative yield is mentioned. Is this the same? It is also unclear what the boxes in the panels mean.

Our response: Many thanks for this comment. It is the same. To enhance clarity without using overly complicated expressions, we now write overall yield in the main text and explain its calculation in the figure legend and in the methods (lines 78, 85, 106, 110-113, 522-530, and 567-575). We now also explain the concept of boxplots in the figure legend, as suggested by the reviewer (lines 117-119).

2. Line 106-107: "weak bottlenecks consistently favoured variants in only few genes", for which Fig. 2 shows little support, with 5 versus 7 genes affected under Gen selection.
3. Line 131-132: "In consistency with the GEN experiment, weak bottlenecks led to variants in much

fewer genes (Fig. 2b)", again for which Fig. 2b provides little support, because there are no data for comparing bottleneck size, not for IC20 (IC20-M5 data lacking) nor for IC80 where only 1 population is shown for k50 bottleneck.

Our response: Many thanks for these related comments. We agree that we should have been more careful in our wording. We now carefully describe that high population variant frequencies appear to occur for fewer genes under wide bottlenecks in both experiments and also visible in the genomics of the end of the experiment and throughout experimental evolution. See changes in lines 123-126, 133, and 152-162.

4. Fig. 4b: The figure says "Competitive fitness", but instead shows competitor frequency at the end of competition. Why not present easy-to-interpret relative fitness estimates?

Our response: We agree that the title of the panel was potentially misleading and now write "Competitor frequencies". This is what Fig. 4b does show. We similarly adjusted the wording in the figure legend. See changes in lines 255 and following.

Please note that a relative fitness measure is likely confusing for the reader, because competition is set-up between different pairs of strains. Thus, the relative fitness measures of the competitions on the left part of the panel would have a different reference (e.g., the wildtype strain PA14) than those on the far right (which did not include PA14 in the competitions). Our representation ensures that the competitive performance of all considered strains can be assessed across the different competing pairs. Accordingly, our representation allows the reader to grasp directly that the competitive performance of each mutant strain does depend on the respective competitor and also the experimental conditions.

Reviewer #2 (Remarks to the Author):

The manuscript explores the role of population bottlenecks and selection intensity in the evolution of antibiotic resistance. The authors employ a novel flow-cytometry method to manipulate bottleneck intensity, and then experimentally evolve resistance to two doses of two antibiotics. The author characterize adaptation at both the phenotypic and genomic scales. The authors find that the evolution of resistance can be favoured by either strong selection and weak bottlenecks, or weak selection and strong bottlenecks. The authors discuss their results in relation to evolutionary theory, published manuscripts, and clinical scenarios.

The manuscript is well written, the methods are clear, and the data visualisations are excellent throughout. However I have reservations about how the authors relate their experiment design and findings to both theory and other experiments.

The problem with this design is that it has decoupled population size and bottleneck intensity. As such both the predictions and the data interpretations are not as straightforward as the authors suggest. The theory the authors cite in the introduction is about population size; big populations have a greater supply

of beneficial mutations, therefore faster, more parallel adaptation. If bottleneck size were the only treatment, this theory could be applied directly, as it would correlate with population size. But as selection intensity also affects population size, the predictive power and relevance of bottleneck size is far less clear. For example, the weak-selection, strong bottleneck treatment (IC20 K50) creates a decent population size and it is not "unexpected" that resistance can evolve quite readily under these conditions. Likewise, the lack of adaptation in the populations with a greater population size experiencing the same weak selection is also not unexpected. The ancestor will be harder to out-compete, as well as the greater mutation supply will open other routes of adaptation, not just evolving resistance.

I am not suggesting that the manuscript is fundamentally flawed, but I would suggest the manuscript would benefit from a more nuanced setup and interpretation. Although the authors do discuss the role of population size at length in the discussion, I feel it needs greater prominence throughout the manuscript.

Our response: This is a very important point and we agree with all concerns. In fact, it is something, which we intensively discussed while setting up the project and designing the experiment. We now realized that we should have explained our rationale in more detail. Our aim was to assess to what extent bottlenecks – which are common during infection – affect the pathogen's ability to respond to selection. As the reviewer correctly points out, antibiotics themselves affect population size. The same applies to any other selective constraint. Therefore, the evolutionary/adaptive consequences of a bottleneck cannot be viewed alone in isolation, but it always needs to be related to the relevant selective constraint that challenges the evolving populations after having or not having experienced the bottleneck. In the end, this is the reason why we included two distinct antibiotic concentrations in our experimental design, because this then allows us to assess whether the bottleneck alone or its interaction with the selective constraint determines the ability of the bacteria to adapt.

We believe that this design is not a problem, but actually a particular strength of our study. By varying both, we can specifically assess which aspects are affected by a strong bottleneck alone. This would not have been possible if we had used a single selective constraint (i.e., only a single antibiotic concentration). Please note that a particular value of our work is that we could consistently identify the evolutionary consequences of a strong bottleneck across two fully independent experiments, highlighting that the results are robust and meaningful.

In the revised manuscript, we now emphasized the particular importance of varying both factors in order to be able to draw conclusions about the influence of bottlenecks on antibiotic resistance evolution in the abstract (lines 12, 14-15, and 25-26), the introduction (lines 39, 43-44, 50-55) and also the discussion (lines 240-246, 347-352, 355-359).

Minor points

- Perhaps the authors could calculate the harmonic mean population size, which would incorporate both population size and bottleneck size. This may make the manuscript tie-in better with what theory would predict and which of their findings are unexpected.

Our response: Many thanks for this excellent suggestion. We now followed the advice and calculated the harmonic mean of cell counts within one season and used this as an integrative measure of population size during one growth season. The calculation is explained in the methods section and the

results shown in Extended Data Figures 1a and 2a. We also summarized the results by calculating the AUC for the harmonic means across time for a particular replicate population and show the results in Extended Data Figure 1b,c and 2b,c. We statistically evaluated the differences among treatments and show the statistical results in Extended Data Table A. Importantly, the results highlight that overall population size in the 4 treatment groups is primarily, albeit not exclusively determined by the differences in bottleneck size. Therefore, we consider our conclusions on the impact of bottlenecks on resistance evolution to be reliable and meaningful. These aspects are now highlighted in the revised manuscript (lines 69-75, 279-281, and 287-290).

- The language of strong vs wide bottlenecks is odd. These are not contradictory terms, and either strong/weak or narrow/wide would be more consistent.

Our response: Many thanks for the suggestion! We have adjusted our terms in the revised manuscript and have now settled for "strong/weak".

- Line 12-14: what is lacking from the literature is the interaction between bottlenecks and selection intensity, and I think this sentence should be rephrased to reflect the manuscripts novelty. In general I think the abstract and introduction could do a better job emphasising this aspect of the work.
- Line 31-33: I think this is a debatable point, so I would change this to "may contribute" or "could be contributing". The citations the authors provide to support this statement discuss the role of environmental contamination but provide no proof of its importance.
- Line 37. "In theory." I think there are enough published, empirical studies to cut that phrase.
- Line 39-41 . Again the interaction is what is missing, as the author cite more than ten papers exploring the role of bottlenecks in adaptation.
Our response: Many thanks for these comments. We followed the reviewer's suggestions to improve our text of the introduction and abstract. See changes in lines 12, 14-17, 32, 37-38, 42-44.

Reviewer #3 (Remarks to the Author):

Interesting and well performed study addressing the impact of bottlenecks imposed by population size and selection levels on the evolutionary paths to antimicrobial resistance to two antibiotic classes in P. aeruginosa model. Some comments for the authors consideration:

1. Population size is assessed by flow cytometry. How does it reflect cell viability?
Our response: Many thanks for this comment. We always used propidium iodide staining on the bacteria prepared for flow cytometry. Thus, the cytometry results, which we give in our manuscript, always refer to the viable cells. We realized that this was not well described, and thus have included this

Decision Letter, second revision:

19th May 2021

Dear Hinrich,

Thank you for submitting your revised manuscript "Bottleneck size and selection level reproducibly impact antibiotic resistance evolution" (NATECOLEVOL-201212451B). It has now been seen again by the original reviewers and their comments are below. The reviewers find that the paper has improved in revision, and therefore we'll be happy in principle to publish it in Nature Ecology & Evolution, pending minor revisions to satisfy the reviewers' final requests and to comply with our editorial and formatting guidelines.

If the current version of your manuscript is in a PDF format, please email us a copy of the file in an editable format (Microsoft Word or LaTex)-- we can not proceed with PDFs at this stage.

We are now performing detailed checks on your paper and will send you a checklist detailing our editorial and formatting requirements in about a week. Please do not upload the final materials and make any revisions until you receive this additional information from us.

Thank you again for your interest in Nature Ecology & Evolution. Please do not hesitate to contact me if you have any questions.

[REDACTED]

Reviewer #1 (Remarks to the Author):

I thank the authors for their response to my comments. I had three main comments, which in my view limited the advance made by this study: (1) the authors use yield instead of more conventional and universally relevant growth rate as measure of fitness, (2) without knowing the cause of the high yield of the 8 CIP-treated IC20/M5 populations, it is unclear how these results should be seen in light of the issue of bottleneck size and selection strength – as the arguments are about mutation supplies and competitive fitness of mutants, and (3) consider mutation bias in the interpretation of the observed effects from bottleneck and selection strength. I am satisfied with the authors' response to my 3rd comment, but not so much with their reaction on the first two issues.

With respect to yield, the authors say that yield can be reliably measured whereas growth rate cannot during initial growth. This may indeed partly justify their focus on yield, but it should then still be explained how yield relates to fitness. The fact that yield and fitness seem to trade off for some treatments, suggest that this relationship is at least complex and requires discussion.

With respect to the lack of explanation of the CIP IC20/M5 results, I remain puzzled about how to interpret these results in terms of effects from bottleneck size and selection strength. Should these findings simply be ignored, or do they speak to the effects from bottlenecks and selection used to

interpret the other results? While it would be best to sort out the cause of the high yield of these populations (e.g. using long-read sequencing to detect rearrangements), the authors should at least discuss what the lack of finding a genetic cause implies for the overall conclusion based on the supply and fitness effects of mutations involved.

Reviewer #2 (Remarks to the Author):

This is a review of the resubmitted manuscript by Mahrt et al. I think the authors have done a good job replying to the reviewer comments and they have made substantial improvements to the manuscript. I think the introduction does a much better job of establishing the question, and consequently the data interpretation is now much clearer. I have only a few minor additional points.

Minor comments
1)Line 123: This paragraph is about the GEN experiment, which needs to be stated at its beginning.
2)Line 298-299: “especially in the GEN evolution experiments, in which genetically manifested evolutionary changes occurred.” Is this an odd way of saying in which mutations could be detected, or evolutionary changes occurred, or other more conventional phrases.
3)Line 517: the carbon source added to the M9 is missing.

Reviewer #3 (Remarks to the Author):

All points raised to the previous version have been satisfactorily addressed and therefore I have no further comments for the authors consideration

Our ref: NATECOLEVOL-201212451B

24th May 2021

Dear Dr. Schulenburg,

Thank you for your patience as we’ve prepared the guidelines for final submission of your Nature Ecology & Evolution manuscript, "Bottleneck size and selection level reproducibly impact antibiotic resistance evolution" (NATECOLEVOL-201212451B). Please carefully follow the step-by-step instructions provided in the attached file, and add a response in each row of the table to indicate the changes that you have made. Please also check and comment on any additional marked-up edits we have proposed within the text. Ensuring that each point is addressed will help to ensure that your revised manuscript can be swiftly handed over to our production team.

We would like to start working on your revised paper, with all of the requested files and forms, as soon as possible (preferably within two weeks). Please get in contact with us immediately if you anticipate it taking more than two weeks to submit these revised files.

When you upload your final materials, please include a point-by-point response to any remaining reviewer comments.

If you have not done so already, please alert us to any related manuscripts from your group that are under consideration or in press at other journals, or are being written up for submission to other journals (see: https://www.nature.com/nature-research/editorial-policies/plagiarism#policy-on-duplicate-publication for details).

In recognition of the time and expertise our reviewers provide to Nature Ecology & Evolution's editorial process, we would like to formally acknowledge their contribution to the external peer review of your manuscript entitled "Bottleneck size and selection level reproducibly impact antibiotic resistance evolution". For those reviewers who give their assent, we will be publishing their names alongside the published article.

Nature Ecology & Evolution offers a Transparent Peer Review option for new original research manuscripts submitted after December 1st, 2019. As part of this initiative, we encourage our authors to support increased transparency into the peer review process by agreeing to have the reviewer comments, author rebuttal letters, and editorial decision letters published as a Supplementary item. When you submit your final files please clearly state in your cover letter whether or not you would like to participate in this initiative. Please note that failure to state your preference will result in delays in accepting your manuscript for publication.

Cover suggestions

As you prepare your final files we encourage you to consider whether you have any images or illustrations that may be appropriate for use on the cover of Nature Ecology & Evolution.

Covers should be both aesthetically appealing and scientifically relevant, and should be supplied at the best quality available. Due to the prominence of these images, we do not generally select images featuring faces, children, text, graphs, schematic drawings, or collages on our covers.

We accept TIFF, JPEG, PNG or PSD file formats (a layered PSD file would be ideal), and the image should be at least 300ppi resolution (preferably 600-1200 ppi), in CMYK colour mode.

If your image is selected, we may also use it on the journal website as a banner image, and may need to make artistic alterations to fit our journal style.

Please submit your suggestions, clearly labeled, along with your final files. We'll be in touch if more information is needed.

Nature Ecology & Evolution has now transitioned to a unified Rights Collection system which will allow our Author Services team to quickly and easily collect the rights and permissions required to publish

your work. Approximately 10 days after your paper is formally accepted, you will receive an email in providing you with a link to complete the grant of rights. If your paper is eligible for Open Access, our Author Services team will also be in touch regarding any additional information that may be required to arrange payment for your article.

Please note that <i>Nature Ecology & Evolution</i> is a Transformative Journal (TJ). Authors may publish their research with us through the traditional subscription access route or make their paper immediately open access through payment of an article-processing charge (APC). Authors will not be required to make a final decision about access to their article until it has been accepted. Find out more about Transformative Journals

<B>Authors may need to take specific actions to achieve compliance with funder and institutional open access mandates. For submissions from January 2021, if your research is supported by a funder that requires immediate open access (e.g. according to Plan S principles) then you should select the gold OA route, and we will direct you to the compliant route where possible. For authors selecting the subscription publication route our standard licensing terms will need to be accepted, including our self-archiving policies. Those standard licensing terms will supersede any other terms that the author or any third party may assert apply to any version of the manuscript.

Please note that you will not receive your proofs until the publishing agreement has been received through our system.

For information regarding our different publishing models please see our Transformative Journals page. If you have any questions about costs, Open Access requirements, or our legal forms, please contact ASJournals@springernature.com.

Please use the following link for uploading these materials:
[REDACTED]

If you have any further questions, please feel free to contact me.

[REDACTED]

Reviewer #1:
Remarks to the Author:
I thank the authors for their response to my comments. I had three main comments, which in my view limited the advance made by this study: (1) the authors use yield instead of more conventional and universally relevant growth rate as measure of fitness, (2) without knowing the cause of the high yield of the 8 CIP-treated IC20/M5 populations, it is unclear how these results should be seen in light of the issue of bottleneck size and selection strength – as the arguments are about mutation supplies

and competitive fitness of mutants, and (3) consider mutation bias in the interpretation of the observed effects from bottleneck and selection strength. I am satisfied with the authors' response to my 3rd comment, but not so much with their reaction on the first two issues.

With respect to yield, the authors say that yield can be reliably measured whereas growth rate cannot during initial growth. This may indeed partly justify their focus on yield, but it should then still be explained how yield relates to fitness. The fact that yield and fitness seem to trade off for some treatments, suggest that this relationship is at least complex and requires discussion.

With respect to the lack of explanation of the CIP IC20/M5 results, I remain puzzled about how to interpret these results in terms of effects from bottleneck size and selection strength. Should these findings simply be ignored, or do they speak to the effects from bottlenecks and selection used to interpret the other results? While it would be best to sort out the cause of the high yield of these populations (e.g. using long-read sequencing to detect rearrangements), the authors should at least discuss what the lack of finding a genetic cause implies for the overall conclusion based on the supply and fitness effects of mutations involved.

Reviewer #2:
Remarks to the Author:
This is a review of the resubmitted manuscript by Mahrt et al. I think the authors have done a good job replying to the reviewer comments and they have made substantial improvements to the manuscript. I think the introduction does a much better job of establishing the question, and consequently the data interpretation is now much clearer. I have only a few minor additional points.

Minor comments
1)Line 123: This paragraph is about the GEN experiment, which needs to be stated at its beginning.
2)Line 298-299: "especially in the GEN evolution experiments, in which genetically manifested evolutionary changes occurred." Is this an odd way of saying in which mutations could be detected, or evolutionary changes occurred, or other more conventional phrases.
3)Line 517: the carbon source added to the M9 is missing.

Reviewer #3:
Remarks to the Author:
All points raised to the previous version have been satisfactorily addressed and therefore I have no further comments for the authors consideration

Author Rebuttal, second revision:

Reponse to reviewers' comments

Reviewers' comments are in grey and italics, while our response is given in blue.

19th May 2021

Dear Hinrich,

Thank you for submitting your revised manuscript "Bottleneck size and selection level reproducibly impact antibiotic resistance evolution" (NATECOLEVOL-201212451B). It has now been seen again by the original reviewers and their comments are below. The reviewers find that the paper has improved in revision, and therefore we'll be happy in principle to publish it in Nature Ecology & Evolution, pending minor revisions to satisfy the reviewers' final requests and to comply with our editorial and formatting guidelines.

If the current version of your manuscript is in a PDF format, please email us a copy of the file in an editable format (Microsoft Word or LaTex)-- we can not proceed with PDFs at this stage.

We are now performing detailed checks on your paper and will send you a checklist detailing our editorial and formatting requirements in about a week. Please do not upload the final materials and make any revisions until you receive this additional information from us.

Thank you again for your interest in Nature Ecology & Evolution. Please do not hesitate to contact me if you have any questions.

Best wishes
Patrick

Patrick Goymer, DPhil
Chief Editor
Nature Ecology & Evolution

Reviewer #1 (Remarks to the Author):

I thank the authors for their response to my comments. I had three main comments, which in my view limited the advance made by this study: (1) the authors use yield instead of more conventional and universally relevant growth rate as measure of fitness, (2) without knowing the cause of the high yield of the 8 CIP-treated IC20/M5 populations, it is unclear how these results should be seen in light of the issue of bottleneck size and selection strength – as the arguments are about mutation supplies and competitive fitness of mutants, and (3) consider mutation bias in the interpretation of the observed effects from bottleneck and selection strength. I am satisfied with the authors' response to my 3rd comment, but not so much with their reaction on the first two issues.

With respect to yield, the authors say that yield can be reliably measured whereas growth rate cannot during initial growth. This may indeed partly justify their focus on yield, but it should then still be explained how yield relates to fitness. The fact that yield and fitness seem to trade off for some treatments, suggest that this relationship is at least complex and requires discussion.

Our response: Many thanks for this important point. We agree that for the GEN experiment, yield and growth rate do not show a simple linear relationship with each other. In the previous version, we already pointed out that "in the IC20-M5 treatment of the GEN experiment, high overall yield coincides with low growth rates, suggesting that the high cell counts are not caused by the rate of replication alone". In response to the reviewer's comment, we now added that the results further suggest that "that these two components of bacterial fitness (i.e., reproductive rate and final population size) do not show a simple linear relationship with each other." We decided against a more detailed discussion of the exact causes of such a more complex relationship, as such a discussion would have to be highly speculative. Instead, we continue to discuss the results of the competition experiments, which were focused on the GEN experiment and did help us explain why certain variants spread under the low selection IC20-M5 conditions and other variants under the more restrictive IC80-M5 conditions. See lines 254 following in the revised manuscript.

With respect to the lack of explanation of the CIP IC20/M5 results, I remain puzzled about how to interpret these results in terms of effects from bottleneck size and selection strength. Should these findings simply be ignored, or do they speak to the effects from bottlenecks and selection used to interpret the other results? While it would be best to sort out the cause of the high yield of these populations (e.g. using long-read sequencing to detect rearrangements), the authors should at least discuss what the lack of finding a genetic cause implies for the overall conclusion based on the supply and fitness effects of mutations involved.

Our response: Many thanks for repeatedly pointing to these interesting results in our data. In response to the reviewer's comments, we now re-wrote the part of the discussion, in which we previously discussed a possible explanation for the lack of any genetic changes under the IC20-M5 treatment in the CIP experiment. In particular, we now acknowledge that one explanation could be that the evolutionary response is due to genomic rearrangements, which cannot be easily detected with the short-read sequencing data obtained for this study. We further point out that an alternative explanation is that purely phenotypic responses are sufficient under these low-selection conditions for the bacteria to survive and proliferate. In response to the reviewer's comment, we now also highlight that this then also means that the phenotypic responses are sufficient for the wildtype strain to outcompete any low-level resistance variants, which we observed to emerge and spread in the GEN experiment under these treatment conditions. In detail, in the revised discussion, we now write:

"...in the CIP experiment, we could not detect any variants spreading under weak bottlenecks combined with low selection levels (i.e., the IC20-M5 treatment), even though populations increased their overall yield (Figure 1c). This result may suggest that the evolutionary response is due to genomic rearrangements, which cannot be easily inferred from the short-read sequencing data obtained for this study. As an alternative explanation, phenotypic responses are sufficient to counter the low selective

constraints imposed, thus allowing the wildtype bacteria to proliferate (Figure 1c) and to outcompete any low resistance variants, which were observed to spread in the GEN experiment under the IC20-M5 conditions (Figures 1a, 2a, 3b, and 4)." See lines 221-229 of the revised manuscript.

Reviewer #2 (Remarks to the Author):

This is a review of the resubmitted manuscript by Mahrt et al. I think the authors have done a good job replying to the reviewer comments and they have made substantial improvements to the manuscript. I think the introduction does a much better job of establishing the question, and consequently the data interpretation is now much clearer. I have only a few minor additional points.

Minor comments
1)Line 123: This paragraph is about the GEN experiment, which needs to be stated at its beginning.

Our response: Many thanks for this comment. The results described in the first sentence do indeed refer to the GEN experiment. As suggested, this is now clarified at the beginning of the sentence where we state that: "Our subsequent genome analysis of the populations from the GEN experiment revealed that..." See line 115 in the revised manuscript.

2)Line 298-299: "especially in the GEN evolution experiments, in which genetically manifested evolutionary changes occurred." Is this an odd way of saying in which mutations could be detected, or evolutionary changes occurred, or other more conventional phrases.

Our response: Many thanks for the comment and apologies for odd expressions. We attempted to emphasize here that evolutionary changes (which by definition must be genetically manifested) occurred across all treatments of the GEN experiment. To avoid confusion, we now re-wrote this part of the sentence and say: "..., especially in the GEN evolution experiments, for which this treatment consistently resulted in evolutionary changes (confirmed by the observed genetic changes, Figures 2 and 3)." See lines 251-254 of the revised manuscript.

3)Line 517: the carbon source added to the M9 is missing.

Our response: In the revised methods, we now provide details of the composition of the M9 medium and the added carbon source (in our case, this was glucose). See lines 540-542 in the revised manuscript.

Reviewer #3 (Remarks to the Author):

All points raised to the previous version have been satisfactorily addressed and therefore I have no further comments for the authors consideration

Our response: Many thanks!

natureresearch

Final Decision Letter:

11th June 2021

Dear Professor Schulenburg,

We are pleased to inform you that your Article entitled "Bottleneck size and selection level reproducibly impact antibiotic resistance evolution", has now been accepted for publication in Nature Ecology & Evolution.

Before your manuscript is typeset, we will edit the text to ensure it is intelligible to our wide readership and conforms to house style. We look particularly carefully at the titles of all papers to ensure that they are relatively brief and understandable.

The subeditor may send you the edited text for your approval. Once your manuscript is typeset you will receive a link to your electronic proof via email, with a request to make any corrections within 48 hours. If you have queries at any point during the production process then please contact the production team at rjsproduction@springernature.com. Once your paper has been scheduled for online publication, the Nature press office will be in touch to confirm the details.

Acceptance of your manuscript is conditional on all authors' agreement with our publication policies (see www.nature.com/authors/policies/index.html). In particular your manuscript must not be published elsewhere and there must be no announcement of the work to any media outlet until the publication date (the day on which it is uploaded onto our web site).

Please note that <i>Nature Ecology & Evolution</i> is a Transformative Journal (TJ). Authors may publish their research with us through the traditional subscription access route or make their paper immediately open access through payment of an article-processing charge (APC). Authors will not be required to make a final decision about access to their article until it has been accepted. Find out more about Transformative Journals

<B>Authors may need to take specific actions to achieve compliance with funder and institutional open access mandates. For submissions from January 2021, if your research is supported by a funder that requires immediate open access (e.g. according to Plan S principles) then you should select the gold OA route, and we will direct you to the compliant route where possible. For authors selecting the subscription publication route our standard licensing terms will need to be accepted, including our self-archiving policies. Those standard licensing terms will supersede any other terms that the author or any third party may assert apply to any version of the manuscript.

In approximately 10 business days you will receive an email with a link to choose the appropriate publishing options for your paper and our Author Services team will be in touch regarding any additional information that may be required.

You will not receive your proofs until the publishing agreement has been received through our system.

If you have any questions about our publishing options, costs, Open Access requirements, or our legal forms, please contact ASJournals@springernature.com

An online order form for reprints of your paper is available at https://www.nature.com/reprints/author-reprints.html. All co-authors, authors' institutions and authors' funding agencies can order reprints using the form appropriate to their geographical region.

We welcome the submission of potential cover material (including a short caption of around 40 words) related to your manuscript; suggestions should be sent to Nature Ecology & Evolution as electronic files (the image should be 300 dpi at 210 x 297 mm in either TIFF or JPEG format). Please note that such pictures should be selected more for their aesthetic appeal than for their scientific content, and that colour images work better than black and white or grayscale images. Please do not try to design a cover with the Nature Ecology & Evolution logo etc., and please do not submit composites of images related to your work. I am sure you will understand that we cannot make any promise as to whether any of your suggestions might be selected for the cover of the journal.

You can now use a single sign-on for all your accounts, view the status of all your manuscript submissions and reviews, access usage statistics for your published articles and download a record of your refereeing activity for the Nature journals.

To assist our authors in disseminating their research to the broader community, our SharedIt initiative provides you with a unique shareable link that will allow anyone (with or without a subscription) to read the published article. Recipients of the link with a subscription will also be able to download and print the PDF.

You can generate the link yourself when you receive your article DOI by entering it here: <a href="http://authors.springernature.com/share">http://authors.springernature.com/share<a>.

[REDACTED]

P.S. Click on the following link if you would like to recommend Nature Ecology & Evolution to your librarian http://www.nature.com/subscriptions/recommend.html#forms

** Visit the Springer Nature Editorial and Publishing website at www.springernature.com/editorial-and-publishing-jobs for more information about our career opportunities. If you have any questions please click here.**
